# A randomised controlled trial of heavy shoulder strengthening exercise in patients with hypermobility spectrum disorder or hypermobile Ehlers-Danlos syndrome and long-lasting shoulder complaints: study protocol for the Shoulder-MOBILEX study

**DOI:** 10.1186/s13063-020-04892-0

**Published:** 2020-12-01

**Authors:** Behnam Liaghat, Søren T. Skou, Jens Søndergaard, Eleanor Boyle, Karen Søgaard, Birgit Juul-Kristensen

**Affiliations:** 1 Research Unit for Musculoskeletal Function and Physiotherapy, Department of Sports Science and Clinical Biomechanics, University of Southern Denmark, Campusvej 55, 5230 Odense, Denmark; 2 Department of Physiotherapy and Occupational Therapy, Næstved-Slagelse-Ringsted Hospitals, Slagelse, Denmark; 3 Research Unit of General Practice, Department of Public Health, Faculty of Health Sciences, University of Southern Denmark, Odense, Denmark; 4 Research Unit of Physical Activity and Health in Work Life, Department of Sports Science and Clinical Biomechanics, University of Southern Denmark, Odense, Denmark; 5 Department of Clinical Research, University of Southern Denmark, Odense, Denmark

**Keywords:** Hypermobility, Hypermobility spectrum disorder, Hypermobile Ehlers-Danlos syndrome, Shoulder, Strength, WOSI

## Abstract

**Background:**

Four out of five patients with hypermobility spectrum disorder (HSD) or hypermobile Ehlers-Danlos syndrome (hEDS) experience shoulder complaints including persistent pain and instability. Evidence suggests that patients with HSD/hEDS who experience knee and back complaints improve with exercise-based therapy. However, no study has focused on exercise-based treatment for the shoulder in this patient group. The potential benefits of strengthening the shoulder muscles, such as increased muscle-tendon stiffness, may be effective for patients with HSD/hEDS who often display decreased strength and increased shoulder laxity/instability.

The primary aim is to investigate the short-term effectiveness of a 16-week progressive heavy shoulder strengthening programme and general advice (HEAVY) compared with low-load training and general advice (LIGHT), on self-reported shoulder symptoms, function, and quality of life.

**Methods:**

A superiority, parallel group, randomised controlled trial will be conducted with 100 patients from primary care with HSD/hEDS and shoulder complaints (persistent pain and/or instability) for more than 3 months. Participants will be randomised to receive HEAVY (full range of motion, high load) or LIGHT (neutral to midrange of motion, low load) strengthening programme three times weekly with exercises targeting scapular and rotator cuff muscles. HEAVY will be supervised twice weekly, and LIGHT three times during the 16 weeks. The primary outcome will be between-group difference in change from baseline to 16-week follow-up in the Western Ontario Shoulder Instability Index (WOSI, 0-2100 better to worse). Secondary outcomes will include a range of self-reported outcomes covering symptoms, function, and quality of life, besides clinical tests for shoulder strength, laxity/instability, and proprioception. Outcome assessors will be blinded to group allocation. Participants will be kept blind to treatment allocation through minimal information about the intervention content and hypotheses. Primary analyses will be performed by a blinded epidemiologist.

**Discussion:**

If effective, the current heavy shoulder strengthening programme will challenge the general understanding of prescribing low-load exercise interventions for patients with HSD/hEDS and provide a new treatment strategy. The study will address an important and severe condition using transparent, detailed, and high-quality methods to potentially support a future implementation.

**Trial registration:**

ClinicalTrials.gov NCT03869307. Registered on 11 March 2019.

**Supplementary information:**

The online version contains supplementary material available at 10.1186/s13063-020-04892-0.

## Administrative information

Note: the numbers in curly brackets in this protocol refer to SPIRIT checklist item numbers. The order of the items has been modified to group similar items (see http://www.equator-network.org/reporting-guidelines/spirit-2013-statement-defining-standard-protocol-items-for-clinical-trials/).

**Table:** 

Title {**1}**	A randomised controlled trial of heavy shoulder strengthening exercise in patients with Hypermobility Spectrum Disorder (HSD)/hypermobile Ehlers Danlos Syndrome (hEDS) and long-lasting shoulder complaints: study protocol for the Shoulder-MOBILEX study
Trial registration {**2a and 2b}**	ClinicalTrials.gov Identifier: NCT03869307. The register collects all items from the World Health Organization Trial Registration Data Set.
Protocol version {**3}**	Issue date: 6 Apr 2020 Amendment 2.
Funding {**4}**	This work is supported by the Region of Southern Denmark, Esbjerg municipality, The Danish Rheumatism Association, Fund for Research, Quality and Education in Physiotherapy Practice, and University of Southern Denmark, all in Denmark. STS is currently funded by a grant from Region Zealand in Denmark (Exercise First) and a grant from the European Research Council (ERC) under the European Union’s Horizon 2020 Research and Innovation Program (grant agreement No. 801790).
Author details {**5a}**	Research Unit for Musculoskeletal Function and Physiotherapy, Department of Sports Science and Clinical Biomechanics, University of Southern Denmark, Odense, Denmark. Department of Physiotherapy and Occupational Therapy, Næstved-Slagelse-Ringsted Hospitals, Næstved, Slagelse and Ringsted, Denmark. Research Unit of General Practice, Department of Public Health, Faculty of Health Sciences, University of Southern Denmark, Odense, Denmark. Research Unit of Physical Activity and Health in Work Life, Department of Sports Science and Clinical Biomechanics, University of Southern Denmark, Odense, Denmark. Department of Clinical Research, University of Southern Denmark, Odense, Denmark.
Name and contact information for the trial sponsor {**5b}**	The study sponsor is Research Unit for Musculoskeletal Function and Physiotherapy, Department of Sports Science and Clinical Biomechanics, University of Southern Denmark, Odense, Denmark. Campusvej 55, 5230, Odense, Denmark, where the project is being conducted/hosted.
Role of sponsor {**5c}**	Associate Professor Birgit Juul-Kristensen took part in designing the study and is a member of the Steering Committee.

## Introduction

### Background and rationale {6a}

Generalised joint hypermobility (GJH) is a hereditary condition characterised by an increased ability to move the joints beyond the normal range of motion. The prevalence in the general population is up to 57% depending on race, sex, and diagnostic criteria [1, 2]. Often, GJH is non-symptomatic and may be recognised as an advantage in many activities, especially in sports where high flexibility is required [3]. On the contrary, GJH may be symptomatic with chronic/recurrent pain, joint instability, musculoskeletal problems, fatigue, and disability, meaning a decreased ability to participate in daily activities, poor health-related quality of life, and increased psychological problems [3–7]. Four out of five people with GJH experience symptoms in the shoulder joint [3, 8–10].

Hypermobility spectrum disorder (HSD) is a recently defined group of conditions related to GJH, including one or more secondary symptomatic musculoskeletal manifestations [11]. The HSD criteria are similar to those of the rare genetic connective tissue disorder, hypermobile Ehlers-Danlos syndrome (hEDS), but without fully meeting the new diagnostic criteria for hEDS (e.g. signs of faulty connective tissue throughout the body including skin features, hernias, prolapses). Although patients with HSD/hEDS and shoulder complaints can experience profound consequences in daily living [4, 5], there is no gold standard treatment for this patient group. They often receive a non-standardised treatment consisting of a combination of physiotherapy modalities including low-dose exercise prescription based on limited evidence [12, 13]. As suggested from few randomised controlled trials (RCT) and uncontrolled studies without long-term follow-up including unclear exercise descriptions, patients with HSD/hEDS may improve with exercise-based therapy. However, specific exercise types are not suggested [12–15], and no study has focused on treatment of the shoulder in this patient group.

An important component of exercise interventions for long-lasting shoulder complaints, such as rotator cuff tendinopathy and anterior and multidirectional glenohumeral instability, is strength training aimed at strengthening the scapular stabilising muscles and rotator cuff muscles [16–18]. Mechanical loading is known to increase muscle strength and tendon stiffness [19, 20], which may be valuable for treatment of patients with HSD/hEDS, as they often display general strength impairments in the shoulder [21]. Therefore, the positive effects of progressive heavy strength training, such as increased muscle-tendon stiffness and improved physical function, may benefit patients with HSD/hEDS.

Many clinicians hesitate to use heavy strengthening exercise for patients with HSD/hEDS, due to uncertainty about patient safety, and because to date, there have not been any RCTs that have investigated the effectiveness of heavy strengthening exercise in adults with HSD/hEDS and shoulder complaints. However, our recent feasibility study on 12 patients regarding (i) patient recruitment and retention, (ii) adherence to exercise protocol, (iii) completion of testing protocol, and (iv) number of adverse events showed that patients could safely complete a 16-week progressive heavy shoulder strengthening programme [22]. Moreover, there were apparent clinical benefits in self-reported shoulder function and objective measurements, suggesting that patients with HSD/hEDS and shoulder complaints benefit from a heavy strengthening programme. This justifies the need for a thorough evaluation in an RCT.

### Objectives {7}

#### Primary research question

The research question for this study is: “What is the effectiveness of a 16-week progressive heavy shoulder strengthening exercise programme and general advice (HEAVY) compared with a low-load shoulder exercise programme and general advice (LIGHT) (current standard care in Denmark) for improving self-reported shoulder function 16 weeks after baseline, measured by using the Western Ontario Stability Index (WOSI), in patients with HSD/hEDS and long-lasting shoulder complaints (persistent pain or instability for more than 3 months) seeking primary care?”

The research question was framed using the PICOT model [23]:

- Population: patients with HSD/hEDS and long-lasting shoulder complaints (persistent pain or instability for more than 3 months)
- Intervention: heavy shoulder strengthening exercises and general advice (HEAVY)
- Control: low-load shoulder exercises and general advice (LIGHT)
- Outcome: self-reported shoulder function using WOSI
- Timeframe: primary endpoint 16 weeks after baseline.

The study acronym is the **shoulder** hyper**mobil**ity **ex**ercise study (The Shoulder-MOBILEX study).

#### Primary objective

The primary objective is to investigate the between-group difference in the change score of HEAVY versus LIGHT on self-reported shoulder-related symptoms, function and quality of life from baseline to the primary endpoint at 16-week follow-up (short-term effectiveness).

#### Primary research hypothesis

The primary hypothesis is that HEAVY is superior to LIGHT in terms of reducing shoulder complaints, including pain, as well as increasing self-reported shoulder function and quality of life. These effects will also lead to a lower level of disability with potential for increased participation in leisure time activities. Furthermore, it is hypothesised that HEAVY will increase muscle strength and shoulder proprioception and decrease shoulder laxity more than LIGHT.

#### Secondary objectives

The secondary objectives are to assess the between-group difference in the change score in HEAVY and LIGHT from baseline to 12-month follow-up (long-term effectiveness) on the same parameters as in the short-term effectiveness and to report the clinical characteristics of the participants at baseline and at 16-week and 12-month follow-up.

### Trial design {8}

**Fig. 1:**
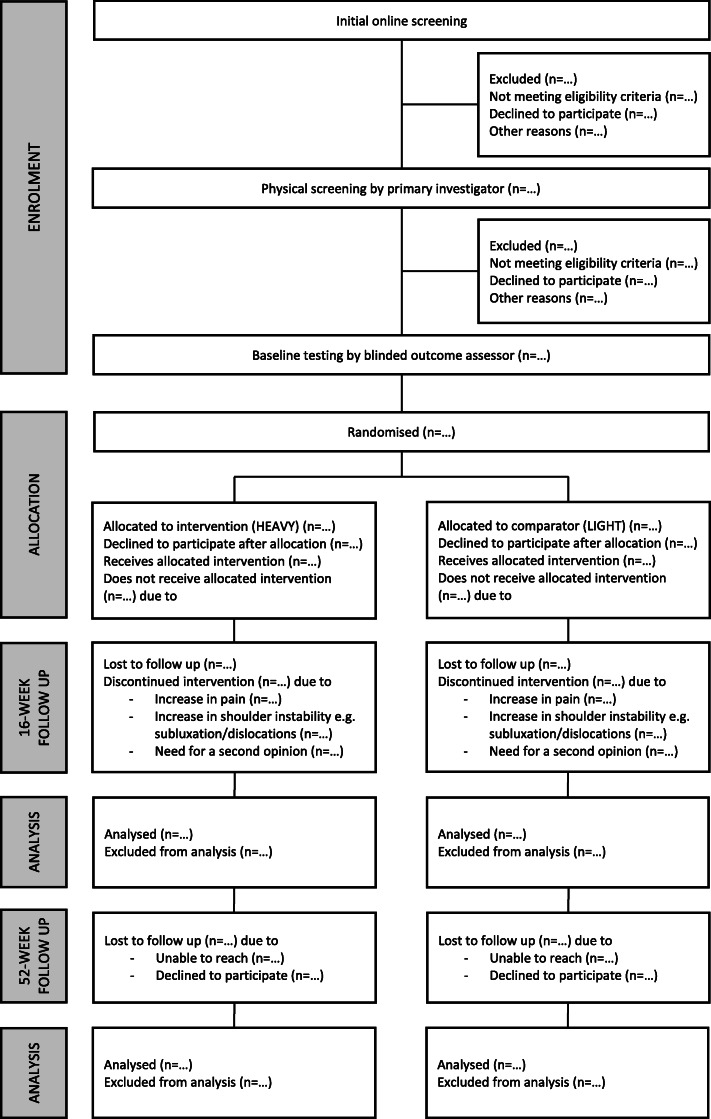
Participant flow chart

This study is an assessor-blinded, multicentre, randomised, controlled superiority trial with a two-group parallel design, comparing HEAVY with LIGHT (considered standard care in Denmark) (Fig. 1). Participants will be randomised with a 1:1 allocation ratio, without an option to cross over. The primary endpoint will be the between-group difference in change in self-reported shoulder function from baseline to 16-week follow-up.

This study protocol is based on the PREPARE Trial guide [24] and the SPIRIT checklist [25]. The study report will adhere to the CONSORT guidelines for reporting parallel-group randomised trials. The interventions will be reported according to the TIDieR template for intervention description and replication [26] (Supplementary file 1), the Consensus on Exercise Reporting Template (CERT) [27] (Supplementary file 2), and a mechano-biological description of the exercise programme [28].

## Methods: participants, interventions, and outcomes

### Study setting {9}

This study is conducted in primary care within the Region of Southern Denmark, representing a general patient population in Denmark. Patients will be asked to answer an online pre-screening questionnaire including the five-part questionnaire (5PQ) [26] and questions about shoulder symptoms through the web-based Research Electronic Data Capture (REDCap) [27]. The principal investigator will contact potentially eligible patients with shoulder symptoms for a physical screening using the Beighton tests [29] to make a clinical diagnosis of HSD/hEDS. A project manager at the University of Southern Denmark is responsible for randomisation procedures and practical management of the project. The baseline and follow-up assessments will be completed either at Esbjerg Municipality Rehabilitation Centre, Esbjerg, Denmark, or at the Department of Sports Science and Clinical Biomechanics, University of Southern Denmark, Odense, Denmark, by one of four blinded physiotherapists. The study interventions will be delivered at a physiotherapy clinic close to the participants’ home by one of 23 treating physiotherapists, and the project will cover all treatment expenses. Home-based exercises will take place with no instructor and outside the physiotherapy clinics (e.g. in the participant’s home).

### Eligibility criteria {10}

Patients with HSD/hEDS and shoulder complaints will be included based on the following pre-defined eligibility criteria.

#### Inclusion criteria

Males and females aged between 18 and 65 years fulfilling the following inclusion criteria:

- Generalised HSD (G-HSD) defined using a Beighton score cut off ≥ 5/9 for females up to the age of 50 years, and ≥ 4/9 for those > 50 years and all males [29], or Historical HSD (H-HSD) if the Beighton score is 1 point below the age and sex-specific cut off and the 5PQ is positive (≥ 2/5 positive answers) [30]. Although the shoulder is not assessed in the Beighton score, being classified with GJH by using the Beighton score builds on the assumption that all joints, including the shoulder, are hypermobile. Therefore, no additional tests for shoulder hypermobility will be used as part of the inclusion criteria.
- One or more of the following self-reported (yes/no) symptomatic musculoskeletal manifestations present [11, 29]:
  - Musculoskeletal pain in at least one shoulder for at least 3 months.
  - Recurrent joint dislocations or joint instability without a reported history of trauma defined as (a) a minimum of three atraumatic dislocations in the same shoulder, (b) a minimum of two atraumatic dislocations in two different joints (a minimum of one in the shoulder) occurring at different times, and/or (c) medical confirmation of joint instability in at least two joints (a minimum of one in the shoulder).

#### Exclusion criteria

Patients will be excluded if they fulfil any of the following:

- Clinically suspected referred pain from the cervical spine.
- Diagnosis of systemic inflammatory rheumatic diseases, connective tissue diseases (e.g. Marfans, Stickler’s or Loeys Dietz syndromes, EDS except hEDS), and/or neurological diseases.
- Pregnancy or childbirth within the past year or planning to get pregnant during the study period, because of increased levels of relaxin.
- Shoulder surgery within the past year.
- Steroid injection in the affected shoulder in the previous 3 months.
- Inability to speak or understand Danish.
- Inability to comply with the study protocol.
- Inability to provide informed consent.

### Who will provide informed consent? {26a}

The patients will receive oral and written information about the purpose of this study, the study process, and potential risks and benefits. Patient information material, the informed consent form, and a leaflet about patients’ rights in research projects will be delivered to the patient by email from REDCap prior to the physical screening and baseline testing to give the patients time to read, understand, and carefully consider questions they want answered before giving consent to participate. The principal investigator will provide the oral information and answer any questions of clarification regarding the study. The rights of the participants will be protected, and all patients will participate on a voluntary basis and be informed that they can always withdraw from the study without consequences for their subsequent treatment. Voluntary written informed consent will be given to the principal investigator before the physical screening and before the baseline testing session.

### Additional consent provisions for collection and use of participant data and biological specimens {26b}

No ancillary studies are planned.

## Interventions

### Explanation for the choice of comparators {6b}

Despite the high prevalence of shoulder complaints in patients with HSD/hEDS, there is no consensus about which treatment should be offered. The rationale for HEAVY is to target the active elements that are involved in shoulder stability by impacting the cross-sectional areas of the muscles and the voluntary activation of the available muscle mass. Both factors would potentially increase muscle strength after 16 weeks and thereby increase the possibility of establishing active support of the joint to compensate for the lack of passive joint stability in this patient group. We will use an active comparator based on current clinical recommendations, consisting of joint protection advice, prescription of exercises with low load, and education about the condition from the physiotherapist [15, 31, 32]. An important reason for using an active comparator is that we will include patients who have sought medical help by their own initiative, which makes it unethical to offer them a placebo or no treatment (e.g. wait and see), when current available guidelines recommend an active exercise-based approach. Offering a placebo or no treatment may be a potential barrier to study participation, because the possibility of being allocated a placebo-only or no treatment may be perceived as being less desirable than the intervention [33]. Comparing the intervention with another exercise-based treatment, including relevant exercises from previous studies [16–18], will lessen the likelihood of overestimating the clinical effectiveness of the intervention. However, the number of supervised sessions will be much higher in the HEAVY group, potentially introducing more contextual effects and attention-bias that we cannot account for in this study.

### Intervention description {11a}

#### Treating physiotherapists

The treating physiotherapists responsible for delivering the interventions have undergone a 3-h practical and theoretical education programme, supported by an exercise manual provided by the principal investigator (Supplementary files 3 and 4). The same treating physiotherapists will deliver exercise programmes for both groups, and they will be instructed to treat all participants with the same degree of rigour, enthusiasm and optimism. An envelope with exercise manuals for the participant and the physiotherapist will be delivered to every participant to prevent the treating physiotherapists from giving exercises to the wrong group by mistake. The same physiotherapist will supervise the participant through the intervention period, and if needed due to absence, another physiotherapist from the same physiotherapy clinic will take over. In vacation periods, participants will be encouraged to exercise on their own three times weekly, with adjustable dumbbells provided by the project team to the HEAVY group.

#### HEAVY (intervention)

Participants randomised to the intervention group will receive 16 weeks of HEAVY, twice a week (individually supervised) at a physiotherapy clinic (60 min for first session, 30 min for following sessions) and once a week (non-supervised) at home/self-selected location (Table 1, Supplementary files 1–3). The exercise programme includes five exercises for scapular and rotator cuff muscles [16, 34] using custom-made adjustable 3D-printed dumbbells (0–1000 g) and regular dumbbells (2–15 kg): side lying external rotation (ER) in neutral, prone horizontal abduction, prone ER at 90° of shoulder abduction, supine scapular protraction, and seated shoulder elevation in the scapular plane. We use few and basic exercises including simple movements of the shoulder, because the participants have little experience with heavy strengthening exercises, and because we want to focus on the progression in the mechano-biological parameters, i.e. high exercise intensity to achieve great effect on muscle and tendon hypertrophy and strength. At the first session, a 5-repetition maximum (RM) test will be carried out to estimate the 10 RM using Brzycki’s formula [35]. The first 3 weeks consist of a familiarisation period progressing from three sets of a load of 50% of 10 RM in week one, to 70% of 10 RM in the second week and to 90% of 10 RM in the third week. The following 6 weeks (weeks 4–9) will include three sets of 10 RM, and from weeks 10–15, the training load will be four sets of 8 RM [36, 37]. A tapering period will be applied in week 16 to allow for the anabolic response prior to follow-up testing. Each exercise session will consist of 5 min of warm up (performing the exercises unloaded), and participants will receive education in scapular correction and general advice on joint protection adapted by the Danish Rheumatism Association [31] (Supplementary file 3).

**Table 1:** Mechano-biological description of the progressive heavy shoulder strengthening programme (HEAVY) [28]

Week	X_**1**_	X_**2**_	X_**3**_	X_**4**_	X_**5**_	X_**6**_	X_**7**_	X_**8**_	X_**9**_	X_**10**_	X_**11**_	X_**12**_	X_**13**_
1	50% 10 RM	10	3	60 s	3 per week	1 week	3 s shortening 0 s isometric 3 s lengthening	0 s	60 s	No	Full ROM	48 h	Yes
2	70% 10 RM	10	3	60 s	3 per week	1 week	3 s shortening 0 s isometric 3 s lengthening	0 s	60 s	No	Full ROM	48 h	Yes
3	90% 10 RM	10	3	60 s	3 per week	1 week	3 s shortening 0 s isometric 3 s lengthening	0 s	60 s	No	Full ROM	48 h	Yes
4	10 RM	10	3	60 s	3 per week	6 weeks	3 s shortening 0 s isometric 3 s lengthening	0 s	60 s	Yes	Full ROM	48 h	Yes
10	8 RM	8	4	90 s	3 per week	6 weeks	3 s shortening 0 s isometric 3 s lengthening	0 s	48 s	Yes	Full ROM	48 h	Yes
16	70% 8 RM	8	4	90 s	3 per week	1 week	3 s shortening 0 s isometric 3 s lengthening	0 s	48 s	No	Full ROM	48 h	Yes

*X*_*1*_, load magnitude, repetition maximum (RM); *X*_*2*_, number of repetitions; *X*_*3*_, number of sets; *X*_*4*_, rest in-between sets; *X*_*5*_, number of sessions per week; *X*_*6*_, duration of the experimental period; *X*_*7*_, fractional and temporal distribution of the contraction modes per repetition and duration of one repetition; *X*_*8*_, rest in between repetitions; *X*_*9*_, time under tension (s); *X*_*10*_, volitional muscular failure; *X*_*11*_, range of motion (ROM); *X*_*12*_, recovery time in between exercise sessions; *X*_*13*_, predefined anatomical exercise form

#### LIGHT (comparator)

Participants randomised to LIGHT will receive 16 weeks of home-based exercise to be performed in their own home/self-selected location (Table 2, Supplementary files 1–2, 4). Participants will receive a face-to-face individual introduction to the exercises before initiating the programme, and individual supervision at week 5 and week 11, when they start with new exercises (30 min per session). The exercise programme will include nine exercises for scapular and rotator cuff muscles and tendons [16–18]: phase 1 (isometric), posture correction; phase 2 (isometric), shoulder abduction, shoulder internal and external rotation with 90° flexion at the elbow joint and standing weight-bearing in the shoulders against a table; and phase 3 (dynamic with resistance band), shoulder abduction, shoulder internal and external rotation at 90° flexion at the elbow joint, and four-point kneeling with single arm raising. The first 4 weeks consist of the phase 1 exercise, with a set of 10 repetitions (10 s hold per repetition). The following 6 weeks (weeks 5–10) consist of isometric exercises from phase 2 with two sets of 10 repetitions (2–3 s hold). In weeks 11–13, the exercises will be a combination of phase 2 and phase 3 with one set of 10 repetitions of exercises from each phase, and in weeks 14–16, the exercises will be dynamic from phase 3 with two sets of 10 repetitions. Exercises will be performed with or without a TheraBand resistance band and the load will be managed by the participants in accordance with the written instructions. Participants will receive the same instructions and education as participants randomised to HEAVY about scapular correction and general advice on joint protection adapted by the Danish Rheumatism Association [31] (Supplementary file 4).

**Table 2:** Mechano-biological description of the low load exercise programme (LIGHT) [28]

Week	X_**1**_	X_**2**_	X_**3**_	X_**4**_	X_**5**_	X_**6**_	X_**7**_	X_**8**_	X_**9**_	X_**10**_	X_**11**_	X_**12**_	X_**13**_
1	Isometric load	10	1	0 s	3 per week	4 weeks	10 s isometric	0 s	100 s	No	Neutral	48 h	Yes
5	Isometric load	10	2	30 s	3 per week	6 weeks	2–3 s isometric	0 s	20-30 s	No	Neutral	48 h	Yes
11	Isometric load	10	1	30 s	3 per week	3 weeks	2–3 s isometric	0 s	20-30 s	No	Neutral	48 h	Yes
11	Dynamic, light (yellow) resistance band	10	1	30 s	3 per week	3 weeks	3 s shortening 0 s isometric 3 s lengthening	0 s	60 s	No	Mid-range	48 h	Yes
14	Dynamic, light (yellow) resistance band	10	2	30 s	3 per week	3 weeks	3 s shortening 0 s isometric 3 s lengthening	0 s	60 s	No	Mid-range	48 h	Yes

*X*_*1*_, load magnitude; *X*_*2*_, number of repetitions; *X*_*3*_, number of sets; *X*_*4*_, rest in-between sets; *X*_*5*_, number of sessions per week; *X*_*6*_, duration of the experimental period; *X*_*7*_, fractional and temporal distribution of the contraction modes per repetition and duration of one repetition; *X*_*8*_, rest in between repetitions; *X*_*9*_, time under tension; *X*_*10*_, volitional muscular failure; *X*_*11*_, range of motion; *X*_*12*_, recovery time in between exercise sessions; *X*_*13*_, predefined anatomical exercise form

### Criteria for discontinuing or modifying allocated interventions {11b}

The exercise load will be continuously adjusted to the increased or decreased capabilities of the participants: for HEAVY, the load will increase whenever the participant is able to complete more than the pre-defined repetitions for all sets with acceptable symptoms below 5/10 on a Numerical Pain Rating Scale (NPRS) and good movement quality (defined as no glenohumeral subluxation and no increased or obvious scapula dyskinesis [38] compared with unloaded movement). For LIGHT, the progression will be applied by changing from isometric to dynamic exercises during the 16-week exercise programme, by increasing the intensity of the pressure in isometric exercises and by adjusting the length of the resistance band, while keeping acceptable symptoms below 5/10 on the NPRS and maintaining good movement quality. For both groups, the same modification rules apply if participants experience symptoms or pain flares above the acceptable threshold: the demands of the exercise will be reduced (i.e. load, range of motion, sets and repetitions, number of weekly exercise sessions, and by excluding provoking exercises), if symptoms or pain experienced during the exercise session persist for more than 2–3 h, e.g. until the next day or the next exercise session. The exercises will then be performed at that level until the symptoms decrease below the threshold of 5/10 on the NPRS. Thereafter, increases in load will follow the progression as initially planned. For participants with symptoms at rest above 5/10 at baseline, no increase in symptoms will be allowed during exercise.

### Strategies to improve adherence to interventions {11c}

In REDCap, a dashboard report will be created for both groups to monitor whether the weekly email questionnaire has been answered, as well as evaluating exercise adherence in a question regarding number of completed sessions. In case participants fail to attend a supervised exercise session, the treating physiotherapists will contact them by phone to inform them about the importance of adherence to the protocol. Further efforts will be made to make sure that exercise sessions are scheduled as convenient as possible for participants. If participants fail to complete their exercise sessions for 2 consecutive weeks, they will receive an email with follow-up questions about reasons for non-adherence. Every week, the project manager will briefly look at answers from the weekly questionnaire to make sure there are no adverse events preventing the participants from continuing the exercise programme, and if necessary, she will encourage the participants to remember to perform their exercise sessions.

### Relevant concomitant care permitted or prohibited during the trial {11d}

Participants can take pain medication during the study and continue with existing medical treatments as advised by their general practitioner. Participants will be encouraged to limit concomitant interventions for the shoulder such as manual therapy and other physiotherapy treatments. However, if the participant finds any supplementary treatment necessary to be able to complete the current interventions (e.g. manual treatment for exercise-induced headache), they will be encouraged to receive as few of those treatments as possible and report these concomitant interventions on the weekly questionnaire.

### Provisions for post-trial care {30}

No post-trial care will be provided to the participants. However, participants are not restricted from getting other treatments after the 16-week intervention period.

### Outcomes {12}

#### Primary outcome measure

The primary outcome is self-reported shoulder function measured using the WOSI total score developed for patients with shoulder instability [39]. The main analysis will be conducted for changes from baseline to 16-week follow-up, reported as the difference in mean change between groups. The WOSI has 21 questions, each marked on a scale from 0 to 100, with 0 being the best score (no limitations related to the shoulder) and 100 representing the worst score, with a total ranging from 0 to 2100 points [40]. It consists of four subscales: physical symptoms (10 questions; maximum score of 1000); sports/recreation/work (4 questions; maximum score 400); lifestyle (4 questions; maximum score 400); and emotion (3 questions; maximum score 300). The WOSI is responsive and sensitive to change as well as being a valid questionnaire, with a high test-retest reliability [41]. A Danish-validated digital version will be used [39].

#### Secondary outcome measures

**Fig. 2:**
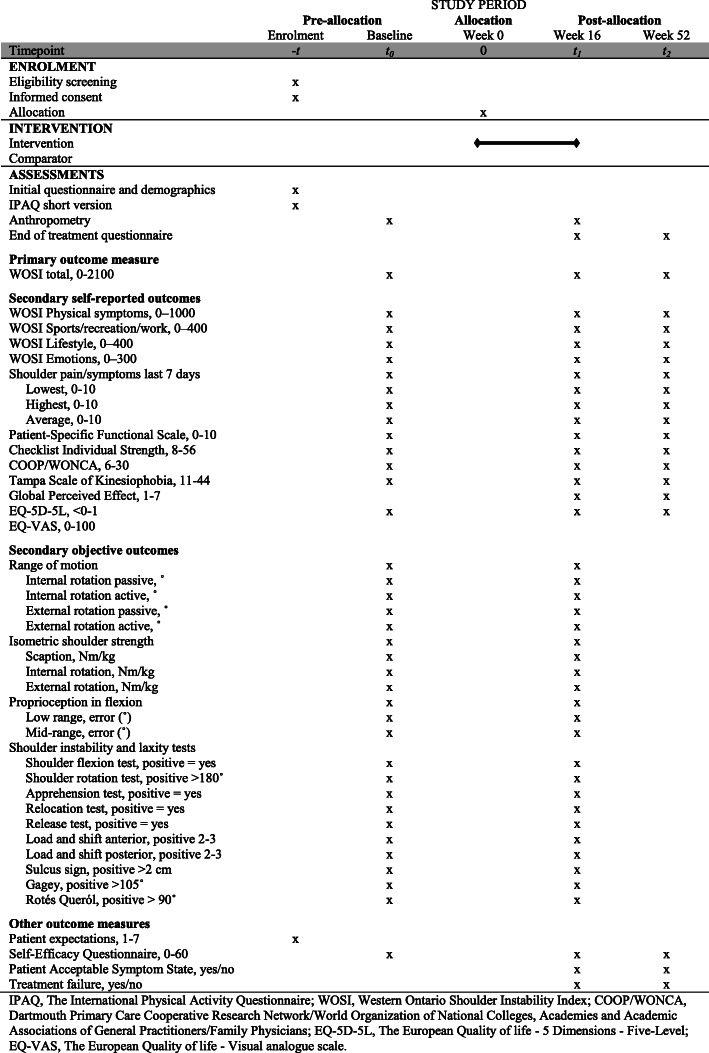
Time schedule of enrolment, interventions, assessments, and visits for participants

Relevant secondary condition-specific and generic self-reported outcomes and objective measurements will be used in this study (Fig. 2). For these outcomes, the main analyses will be conducted for changes from baseline to 16-week follow-up, reported as the difference between groups in mean change or difference in proportions for continuous and dichotomous outcomes, respectively, unless indicated otherwise.

##### Secondary self-reported outcomes

Data from each of the four subscales (physical symptoms, sports/recreation/work, lifestyle, and emotion) from the WOSI questionnaire will be reported. Shoulder pain will be measured using the NPRS with numbers from 0 to 10 (“no pain” to “extreme pain”) [42]. The worst, least, and average pain level for the past week will be measured every week. Pain intensity before and after each exercise session will be measured [42]. Assessment of shoulder symptoms other than pain (instability, subluxation, laxity) will be measured in the same way as for shoulder pain by the NRS from 0 to 10 (“no symptoms” to “extreme symptoms”).

To evaluate whether a health condition impacts the patient’s ability to perform activities they nominate as important to them, the Patient-Specific Functional Scale (PSFS) [43] will be used. At baseline, the participant is asked to identify up to three important activities being difficult or impossible to perform due to symptoms. The participant provides a rating for each selected activity, on an 11-point ordinal scale ranging from 0 (“unable to perform activity”) to 10 (“able to perform activity at the same level as before the injury or problem”). During reassessments, the participant is prompted to re-rate the same activities. The average of up to three specific activity scores is recorded, and the range of possible scores is 0–10. Higher scores indicate less impairment.

Assessment of fatigue will be performed with the Checklist Individual Strength (CIS), the subscale of fatigue, which has shown good reliability and validity. The CIS subscale of fatigue consists of 8 items each scored on a 7-point Likert scale (scores ranging from 8 to 56) with high scores indicating high levels of fatigue [44].

To assess functional health status, the Dartmouth Primary Care Cooperative Research Network/World Organization of National Colleges, Academies and Academic Associations of General Practitioners/Family Physicians (COOP/WONCA) questionnaire will be used. The questionnaire is a generic health status questionnaire for general practice patients. The questionnaire consists of six single-item measures: physical fitness, feelings (mental well-being), daily activities, social activities, change in health, and overall health. The categories chosen are scored from 1 (good functional status) to 5 (poor functional status) with total scores ranging from 6 to 30 [45, 46].

Fear of movement is measured using the Tampa Scale of Kinesiophobia-11 (TSK-11), which consists of an 11-item scale where each question is scored on a 4-point Likert scale, with 1 indicating “strongly disagree” and 4 indicating “strongly agree”. The total scores range from 11 to 44, with higher scores representing increased fear of movement [47].

Health-related quality of life is measured with the generic instrument European Quality of life-5 Dimensions-Five-Level Scale (EQ-5D-5L). It is a classification system that comprises five dimensions (mobility, self-care, usual activities, pain/discomfort and anxiety/depression), where each dimension is rated by a five-level ordinal scale as follows: (1) no problems, (2) slight problems, (3) moderate problems, (4) severe problems, and (5) extreme problems [48]. This classification describes 243 unique health states that are often represented as vectors. From population-based studies, societal value sets have been derived and when they are applied to the health state vectors, they result in a preference-based index that ranges from the state worse than death (< 0), to 1 (full health), with anchoring of death as 0. A score of 1 indicates that the patients perceive their health at the best possible state and a score below 0 that the patients perceive their health worse than death [48, 49]. In addition, the EQ-5D includes an EQ-VAS scale where the patient’s own health “today” is rated between 0 (worst imaginable health) and 100 (best imaginable health). In patients with shoulder instability problems, EQ-5D-5L has satisfactory psychometric properties [49, 50].

To measure the patients’ self-reported impression of important health changes, the Global Perceived Effect (GPE) will be used on each of the four WOSI domains (physical symptoms, sports/recreation/work, lifestyle, and emotions). Patients will rate their experienced change and importance on 7-point scales ranging from “worse, an important worsening” to “better, an important improvement” [51, 52]. The main analysis is conducted to compare the self-reported impression at 16-week follow-up between groups.

##### Secondary objective outcomes

Isometric shoulder strength will be assessed by measuring maximum isometric voluntary contraction in shoulder scaption, internal rotation and external rotation using a handheld dynamometer (IsoForce Dynamometer EVO2; Medical Device Solution AG) [53, 54].

Active and passive shoulder range of motion in internal and external rotation with the shoulder at 90° of abduction [53, 55] and shoulder proprioception in shoulder flexion angles (low-range and mid-range) [56, 57] are assessed using a HALO digital goniometer (Halo Medical Devices, Subiaco, Australia).

Shoulder laxity, hypermobility, and instability will be assessed using the anterior and posterior load and shift [58], sulcus sign [58], Gagey [58], apprehension [58], relocation [58], release [58], Rotés Qúerol [59], and shoulder rotation and flexion [60] tests.

#### Other outcome measures

Participants will be evaluated on self-efficacy related to symptoms, which can be defined as an individual’s confidence to successfully produce desirable results related to living with symptoms. The Self-Efficacy Questionnaire has 10 items which are rated on a 7-point ordinal scale (ranging from 0 “not at all confident”, to 6 “completely confident”). The questionnaire is applicable to patients with persistent pain and covers a range of functions including work, socialising, and household chores as well as coping with pain without medication. The maximum score is 60, and the higher the score, the higher the level of self-confidence managing their symptoms [61].

Participant expectations of treatment effectiveness will be evaluated by asking “How much do you expect your shoulder problem to change as a result of physiotherapy treatment?”, measured on a 7-point Likert scale ranging from 1 “worse than ever” to 7 “complete recovery” [61].

The Patient Acceptable Symptom State (PASS) will be used to evaluate patient satisfaction and consists of simple global dichotomised questions about a patient’s satisfaction of their state of symptoms and treatment failure and is adapted from a previous knee study [62]. Participants will be asked: “When you think of your shoulder function, will you consider your current condition as satisfactory? By shoulder function, you should consider your activities of daily living, sport and recreational activities, your pain and other symptoms, and your quality of life”, with the answer marked by either “yes” or “no”. Participants who answer “no” will be asked to complete the second single-item question, relating to treatment failure: “Would you consider your current state as being so unsatisfactory that you think treatment has failed?”, with the answer marked by “yes” or “no”.

Further, participants will be followed actively by a weekly questionnaire delivered via email as a secure online link with standardised questions about pain and symptoms, amount and type of painkillers, days of being sick or on sick-listing, concomitant treatment during the past week, and potential adverse events.

#### Baseline participant characteristics

General demographic information will include age, sex, civil status, educational level, employment, previous treatment, pain medication, and disease history. Anthropometric measurements include weight and height. To measure physical activity level, the short version of the International Physical Activity Questionnaires (IPAQ) will be used to collect information on time spent in walking and physical activity at moderate and vigorous intensity in four domains: work, transportation, housework/gardening, and leisure time [36, 37]. Participants will be asked to indicate the amount of time spent in physical activity in the past 7 days in relation to frequency (days) and duration (hours or minutes) of the activities.

### Participant timeline {13}

Participants will undergo a structured time schedule including the intervention and assessments (Fig. 2).

### Sample size {14}

Sample size calculation is based on the between-group difference in the mean change scores of WOSI total from baseline to the 16-week follow-up. Based on previous studies on patients with HSD/hEDS, we expect a mean baseline WOSI total of 1050 points [8, 22]. The study will be powered to detect a between-group difference equal to or greater than a previously reported clinically important change of 252 points [40, 63], with an SD for change from baseline to the 16-week follow-up of 350 points [16, 22]. We expect both groups to experience a clinically relevant improvement, seen as a 48% improvement in the HEAVY group (equal to 504 points with an expected baseline mean score of 1050 points) comparable with effects of previous interventions [16, 18, 22, 34, 64, 65] and 24% improvement in the LIGHT group [18] (equal to 252 points with an expected baseline mean score of 1050 points). With a two-sided significance level of 0.05 and 90% power, a sample size of 42 per group is required to detect a statistically significant difference. We decided to enrol 50 patients per group to account for a conservatively estimated dropout of 16% [16, 18]. If 100 patients are not included within 24 months (01 Apr 2019 to 31 Mar 2021), a stopping rule will be applied as soon as a minimum of 76 patients have been included, equal to a power of a minimum of 80%, including 16% dropout.

### Recruitment {15}

Patients will be recruited from April 2019 to March 2021 by general practitioners from local medical clinics and eight physiotherapy clinics in the cities of Odense, Middelfart, and Esbjerg in Denmark. The general practitioners and physiotherapists will receive information about the project, the patient group, and the eligibility criteria to be able to identify and administer the online pre-screening questionnaire to potential patients with HSD/hEDS and long-lasting shoulder complaints in their clinics. All participants had a medical referral to physiotherapy treatment. To achieve adequate enrolment, we have included additional recruitment sites as was apparent to be necessary during the feasibility study as well as social media announcements, and the 2-year recruitment period is anticipated to be adequate time to recruit the required sample size.

## Assignment of interventions: allocation

### Sequence generation {16a}

The allocation sequence will be computer-generated with permuted block randomisation, set up by a data manager outside the project. Participants will be randomly assigned to either HEAVY or LIGHT with a 1:1 allocation ratio.

### Concealment mechanism {16b}

Randomisation is performed automatically in REDCap by the project manager. To ensure allocation concealment, the principal investigator, outcome assessors, and project manager will be blinded to block sizes and unaware of the next assignment in the allocation sequence.

### Implementation {16c}

All eligible patients who give consent for participation and who fulfil the inclusion criteria will be appointed a time for baseline testing. Immediately following the baseline testing session, the project manager will complete the randomisation and reveal the unique group allocation to the patient. Randomisation will be conducted without any influence of the principal investigators, outcome assessors, or treating physiotherapists. The project manager will contact the treating physiotherapist to make sure that all practical aspects run smoothly, including appointment bookings.

## Assignment of interventions: blinding

### Who will be blinded {17a}

The principal investigator and the four outcome assessors will be kept blinded from group allocation.

The participants will be kept blind to treatment allocation by being provided with minimal information about the content of the two exercise interventions and the study hypotheses; the participants will be informed that the study compares two different exercise protocols including safe exercises aimed at increasing the function of the shoulder muscles, but they will not be told of the direction of our hypothesis. During the follow-up testing, participants will be encouraged not to disclose or discuss with the outcome assessors what type of exercises they have performed. The treating physiotherapists responsible for delivering both interventions will not be blinded to which treatment the participants have been allocated to. The treating physiotherapists will be blinded to baseline and follow-up testing results. All pre-defined analyses will be performed by an epidemiologist (EB) blinded to group allocation.

### Procedure for unblinding if needed {17b}

As this study includes two exercise-based interventions with clear modification guidelines related to the participants’ symptom response, emergency unblinding is not relevant. Instead, in case of serious adverse events reported by participants or treating physiotherapists, participants will be referred to their general practitioner, as is the normal procedure in Denmark, and encouraged by the project manager to explain the type and intensity of exercises they have performed.

## Data collection and management

### Plans for assessment and collection of outcomes {18a}

Before starting data collection, the outcome assessors will receive comprehensive education by the principal investigator in the assessment protocol over two sessions, to agree on standardised procedures for all objective outcome measurements. For all participants, data collection will be performed at baseline and at the 16-week follow-up on self-reported outcomes and objective measurements, and at the 12-month follow-up on self-reported outcomes via email as a secure online link (Fig. 2). Participants will complete the self-reported measurements directly into REDCap, and outcome assessors will enter the data in REDCap from their clinical tests and objective examinations at completion of those assessments. Data collection will be performed in an undisturbed room. At supervised exercise sessions, the treating physiotherapists will register the data concerning the exercise session (pain level, load, progressions, repetitions, sets, adverse events, and pain level before and after the session) in a printed exercise logbook, and at home-based sessions, the participants will fill out the exercise logbook. Data from the weekly questionnaire are entered by the participants directly into REDCap. All forms were designed by the Steering Committee.

### Plans to promote participant retention and complete follow-up {18b}

In REDCap, an automatic report has been created to monitor whether the participants answer the weekly questionnaire. For non-responders, an email reminder will be sent out twice with a 1-day interval, and if not answered on the third day, the project manager will contact the participants by phone. Two weeks before the planned 16-week follow-up testing, the project manager will contact the outcome assessor and the participant to make sure that an appointment has been scheduled. The project manager will collect the exercise logbooks regularly as participants complete the 16-week exercise programme.

### Data management {19}

REDCap will be the data collecting and storage system to accomplish the legislative requirements about management and safekeeping of data. A pre-defined codebook is developed, and data will be entered directly into REDCap with validation rules where relevant to verify data entered in a record that meets the specified standards. The principal investigator BL, together with BJK, will have personal access to data by a confidential login to REDCap. An epidemiologist will get access to pseudo-anonymous data in SharePoint or OneDrive. In the “cleaning” process of raw data in preparation for the analysis, the “cleaning” procedures will be saved as a do-file (statistical commands) and data will be saved in a new file to keep the raw data file available. In the process of producing new variables, distribution characteristics of the new variable will be scrutinised and compared with the source and target variable to check for correctness of calculations. A project logbook will be created to be able to keep an overview of management procedures for the data. During data cleaning, data will be checked for duplicates, and summarised data and tabulated data will be used to identify missing data, outliers, and errors. A research assistant outside the project will type in data from the exercise logbooks.

### Confidentiality {27}

All personal information about potential and enrolled patients will be collected and saved in REDCap, which complies with international recommendations for confidential data protection. Medical information about participants in the study will be confidential, and disclosure to third parties other than the Steering Committee (BL, BJK, STS, JS, and KS) will be prohibited. With the participant’s permission, medical information may be shared with his or her personal general practitioner or with other medical personnel responsible for the participant’s care. Data will be de-identified when exported from REDCap. When publishing data from this study, the presentation format will not include names, recognisable photos, personal information, or other data, which may disclose the identity of participants. We will also suppress cell sizes less than three.

### Plans for collection, laboratory evaluation, and storage of biological specimens for genetic or molecular analysis in this trial/future use {33}

Not applicable, as no such samples will be collected.

## Statistical methods

### Statistical methods for primary and secondary outcomes {20a}

The primary analysis will be performed at the primary endpoint (end of the 16-week intervention period). We will prepare a statistical analysis plan and make it publicly available before any analyses commence [66], and report data by using the “CONSORT 2010 statement: updated guidelines for reporting parallel group randomised trials” [67]. All primary analyses will follow the intention-to-treat principle, i.e. all participants will be included in the analysis according to the group to which they were randomised, independent of compliance and withdrawals.

A multivariable linear regression model will be applied to assess the between-group difference in the change from baseline to the 16-week follow-up. The model will contain the primary outcome (change in WOSI total) as the dependent variable, and treatment group (HEAVY or LIGHT) as the main effect, after adjusting for baseline score, age, sex as covariates, and the clustering of physiotherapy clinic. For continuous secondary outcomes, a similar multivariable linear regression model will be applied. For comparison of binary outcomes, multivariable logistic regressions will be applied to estimate between-group difference in proportions at the 16-week follow-up, with outcomes as dependent variables, and treatment group as the main effect, after adjusting for baseline score, age, sex as covariates, and the clustering of physiotherapy clinic.

An alpha level of 0.05 (two-sided) will be considered as being statistically significant. The epidemiologist will be blinded to the allocated interventions for the analysis of the primary outcome. Results for the primary endpoint will undergo blinded interpretation presented in an author consensus statement signed by all authors. The consensus statement will present two versions of the interpretation, first assuming that intervention A is the intervention, and then assuming that intervention B is the intervention [68]. This signed consensus statement will be made publicly available prior to breaking the randomisation code and revealing group allocation. This is to reduce bias and promote transparency in the interpretation of the current findings before any publication procedures are initiated. We will use Stata (StataCorp. 2019. Stata Statistical Software: Release 16. College Station, TX: StataCorp LLC).

### Interim analyses {21b}

Besides the stopping rule defined under the sample size calculation, no other formal stopping guidelines or interim analyses are planned.

### Methods for additional analyses (e.g. subgroup analyses) {20b}

The per-protocol population is defined as participants who adhere to the protocol, defined by the following criteria for both groups: did attend two thirds (67%) or more of the 48 planned exercise sessions, both at supervised clinical sessions and home-based sessions (documented by the exercise logbook and weekly questionnaire); did not stop the intervention during the 16-week intervention period; and did not receive new, important interventions other than the assigned treatment in the follow-up period (e.g. no surgery, no steroid injection, or concomitant supervised exercise-based treatment for the shoulder).

### Methods in analysis to handle protocol non-adherence and any statistical methods to handle missing data {20c}

In the intention-to-treat and per-protocol analyses, missing data at follow-up will be accounted for by using a multiple imputation technique with age, sex, group allocation (masked), and baseline values as predictors. For sensitivity purposes, missing data will be imputed using a non-responder imputation, in which baseline values are carried forward [65]. The rationale builds on the assumption that participants who drop out will return to their baseline WOSI score [66].

### Plans to give access to the full protocol, participant level data, and statistical code {31c}

The full protocol is provided in this paper. Upon publication of the planned research papers, we intend to share the de-identified data for future research purposes on reasonable request.

## Oversight and monitoring

### Composition of the coordinating centre and trial Steering Committee {5d}

BL, BJK, STS, JS, and KS are members of the Steering Committee and are responsible for taking decisions about major changes needed once the study has been initiated. We have no other committees involved in the oversight of the RCT. The principal investigator, BL, checks that recruitment is progressing as necessary and trains the physiotherapists providing the exercise interventions. The project manager checks the data quality and completeness of the data on a weekly basis.

### Composition of the data-monitoring committee, its role, and reporting structure {21a}

The severity of adverse events is expected to be non-critical, and the intervention is not considered a high-risk intervention based on the feasibility study; therefore, a data-monitoring committee will not be established.

### Adverse event reporting and harms {22}

Adverse events will be defined as any unintended, negative findings, symptom, or illnesses that occur during the study assessments or interventions, whether attributable to the project or not. Minor adverse events cover symptom flare up, subluxations, and exercise-induced fatigue. Serious adverse events are unexpected but cover life-threatening events, disability, and permanent damage [69]. Adverse events are recorded at every exercise session in the exercise logbook and in the weekly questionnaire. The treating physiotherapists are familiar with the modification guidelines to reduce the exercise load, if participants experience short-lasting minor adverse events. Acute increase in shoulder symptoms, such as severe shoulder pain (e.g. 8 or higher on the NPRS), including pain during rest or more shoulder subluxations or dislocations than usual, will be reported to the project manager by participants and/or treating physiotherapists. As a safety precaution, if a medical evaluation is indicated, the participants will be referred to their general practitioner, which is the normal procedure in Denmark. Serious adverse events will be reported to the Regional Committee on Health Research Ethics for Southern Denmark within 7 days after the principal investigator or others from the Steering Committee have become aware of the incident without being unblinded. Serious adverse events will be assessed by health professionals outside the project for possible connection with the assessment and/or intervention in the project, but all adverse events will be reported irrespective of their relationship with assessment or intervention. In case of acute injury during the project assessment or intervention, the participants will be able to seek compensation from The Danish Patient Compensations Association and/or by making a complaint to The National Agency for Patients’ Rights and Complaints. We will report both the number of patients experiencing minor adverse events and serious adverse events as well as the number of minor adverse events and serious adverse events during the interventions.

### Frequency and plans for auditing trial conduct {23}

The Regional Committees on Health Research Ethics are annually selecting some studies for auditing. The audit process is independent of investigators and sponsors.

### Plans for communicating important protocol amendments to relevant parties (e.g. trial participants, ethics committees) {25}

Protocol modifications decided by the Steering Committee will be reported to the Regional Committees on Health Research Ethics for Southern Denmark and changes will be added to the ClinicalTrials.gov protocol.

### Dissemination plans {31a}

All results from the study—both positive, negative, and inconclusive—will be published in relevant international scientific peer-reviewed journals. The principal investigator will ensure publication, with authorship following the guidelines of the International Committee of Medical Journal Editors (ICMJE). Results will be presented at relevant national and international conferences, and in relevant patient associations, e.g. the Danish Rheumatism Association and the Hypermobility Association. Prior to publication, a statistical analysis plan will be published online to ensure transparency and high-quality dissemination. The results will be communicated to participants and to the public in general through the media and workshops.

## Discussion

Shoulder complaints are very common in patients with HSD/hEDS, yet current national and international treatment guidelines are based on limited research. More recent systematic reviews have emphasised the need for RCTs investigating the effectiveness of exercise-based treatment in this patient group [12, 13]. The Shoulder-MOBILEX study is the first high-quality multicentre RCT investigating the effectiveness of HEAVY versus LIGHT—the latter considered standard care in Denmark—in patients with HSD/hEDS and shoulder complaints for more than 3 months. The intervention aims at optimising the function of the muscle-tendon unit in the shoulder to provide joint stability and protection, as this is hypothesised to decrease shoulder symptoms, increase function, and improve shoulder-related quality of life in this patient group [19–21, 36]. Studies of other shoulder conditions such as subacromial pain syndrome [70], rotator cuff tendinopathy [17], multidirectional instability [16], and traumatic anterior instability [18] have shown promising results with exercise-based treatment focusing on strengthening the scapular and rotator cuff muscles, suggesting that it might also be effective in the current patient group. If the intervention is found effective, it will potentially be a new treatment option for this patient group, and it can easily be implemented in clinical practice and guide the development of future clinical recommendations.

The research question raised in this study satisfies the FINER criteria [71], as it is considered both feasible, interesting, novel, ethical, and relevant. The study is informed by a high-quality feasibility study that besides finding the intervention, HEAVY, safe and feasible with clinically relevant improvements in shoulder function [22], showed that a few patients experienced adverse events, which were acceptable short-lasting soreness or pain flare-ups. The number of recruitment sites has been increased, as deemed relevant by the feasibility data to complete the RCT successfully. We challenge the general understanding that heavy strengthening exercises should be avoided in this patient group and provide a potentially game-changing alternative to current care. By giving each treatment arm an active exercise-based treatment, where both groups are expected to improve, we will ensure a high ethical standard of research and avoid offering redundant treatments to the patients.

### Strengths and limitations

The LIGHT exercise programme is developed as an active comparator to cover the average exercise-based standard treatment offered across physiotherapy clinics in Danish primary care. Since there is huge variation in treatment among clinics, we could potentially be offering the participants a better or a worse treatment than they would have received outside this project. However, LIGHT is considered superior to wait-and-see or no treatment. It is not within the scope of this study to conduct cost-effectiveness analyses, which could support the most effective exercise programme potentially being implemented, but it could potentially be conducted at a later stage using the national Danish registries.

The pragmatic approach of this study using broad eligibility criteria, a consecutive sampling strategy, standard care as the comparator, and patients recruited from primary care will improve the generalisability of the study results. The pre-registration at ClinicalTrials.gov and publication of this study protocol, including intervention transparency and thoroughly described exercise protocols based on established frameworks, greatly improve the overall quality of the current research study and the potential for implementation.

#### COVID-19 pandemic

The COVID-19 pandemic will inevitably affect the completion of the RCT. The time period for the patient recruitment stopping rule may have to be extended, because recruitment into studies are banned during the measures introduced to manage the pandemic. The treating physiotherapists will be allowed to deliver the intended supervision of exercises via phone and video consultation to manage potential challenges with patient motivation and adherence to the protocol due to quarantine or sickness, and to avoid unnecessary direct physical contact (social distancing). At the 16-week follow-up, the self-reported outcomes, including the WOSI, will be completed via email as a secure online link; the objective measurements will follow as soon as possible, as advised by the Danish Health Authority, and additional sensitivity analyses will be performed on these delayed measurements.

## Trial status

Protocol version

Issue date: 6 Apr 2020.

Protocol amendment 2

Author(s): BL.

Revision chronology

Version 1, 11 Mar 2019 Original.

Version 2, 22 Jul 2019 Amendment 1. Reason for amendment: Steroid injection in the affected shoulder within 3 months was added as an exclusion criterion to make sure that the effect of the injection had worn off.

Version 3, 6 Apr 2020 Amendment 2. Reason for amendment: Typographical error, expected dropout changed from 20 to 16%. Rating scale for GPE specified.

Recruitment

Recruitment was initiated on 01 April 2019 and is expected to be finalised on 31 March 2021, or when a sample size of 100 patients has been achieved.

## Supplementary information

**Additional file 1.** The TIDieR (Template for Intervention Description and Replication) Checklist.

**Additional file 2.** Consensus on Exercise Reporting Template.

**Additional file 3** HEAVY exercise manual.

**Additional file 4.** LIGHT exercise manual.

## Abbreviations

CERT: Consensus on Exercise Reporting Template
CIS: Checklist of Individual Strength
CONSORT: Consolidated Standards of Reporting Trials
COOP/WONCA: Dartmouth Primary Care Cooperative Research Network/World Organization of National Colleges, Academies and Academic Associations of General Practitioners/Family Physicians
EQ-5D-5L: European Quality of life-5 Dimensions-Five-Level
ER: External rotation
GJH: Generalised joint hypermobility
GPE: Global Perceived Effect
hEDS: Hypermobile Ehlers-Danlos syndrome
HSD: Hypermobility spectrum disorder
IPAQ: International Physical Activity Questionnaire
IR: Internal rotation
MCID/C: Minimal clinically important difference/change
NPRS: Numerical Pain Rating Scale
RCT: Randomised controlled trial
REDCap: Research Electronic Data Capture
RM: Repetition maximum
SPIRIT: Standard Protocol Items: Recommendations for Interventional Trials
TSK-11: Tampa Scale of Kinesiophobia-11
TIDieR: Template for Intervention Description and Replication
VAS: Visual Analogue Scale
WOSI: Western Ontario Shoulder Instability Index
5PQ: 5-part Questionnaire

## References

[1] Remvig L, Jensen DV, Ward RC (2007). Epidemiology of general joint hypermobility and basis for the proposed criteria for benign joint hypermobility syndrome: review of the literature. J Rheumatol.

[2] Juul-Kristensen B, Ostengaard L, Hansen S, Boyle E, Junge T, Hestbaek L (2017). Generalised joint hypermobility and shoulder joint hypermobility, - risk of upper body musculoskeletal symptoms and reduced quality of life in the general population. BMC Musculoskelet Disord.

[3] Johnson SM, Robinson CM (2010). Shoulder instability in patients with joint hyperlaxity. J Bone Joint Surg Am.

[4] Mulvey MR, Macfarlane GJ, Beasley M, Symmons DP, Lovell K, Keeley P, Woby S, McBeth J (2013). Modest association of joint hypermobility with disabling and limiting musculoskeletal pain: results from a large-scale general population-based survey. Arthritis Care Res (Hoboken).

[5] Scheper MC, Juul-Kristensen B, Rombaut L, Rameckers EA, Verbunt J, Engelbert RH (2016). Disability in adolescents and adults diagnosed with hypermobility-related disorders: a meta-analysis. Arch Phys Med Rehabil.

[6] Saremi H, Yavarikia A, Jafari N (2016). Generalized ligamentous laxity: an important predisposing factor for shoulder injuries in athletes. Iran Red Crescent Med J.

[7] Easton V, Bacon H, Jerman E, Armon K, Poland F, Macgregor AJ, Smith TO (2014). The relationship between benign joint hypermobility syndrome and psychological distress: a systematic review and meta-analysis. Rheumatology (Oxford).

[8] Johannessen EC, Reiten HS, Lovaas H, Maeland S, Juul-Kristensen B (2016). Shoulder function, pain and health related quality of life in adults with joint hypermobility syndrome/Ehlers-Danlos syndrome-hypermobility type. Disabil Rehabil.

[9] Rombaut L, Malfait F, Cools A, De Paepe A, Calders P (2010). Musculoskeletal complaints, physical activity and health-related quality of life among patients with the Ehlers-Danlos syndrome hypermobility type. Disabil Rehabil.

[10] Palmer S, Cramp F, Lewis R, Gould G, Clark EM (2017). Development and initial validation of the Bristol Impact of Hypermobility questionnaire. Physiotherapy..

[11] Castori M, Tinkle B, Levy H, Grahame R, Malfait F, Hakim A (2017). A framework for the classification of joint hypermobility and related conditions. Am J Med Genet C Semin Med Genet..

[12] Bacon H, Jerman E, Easton V, Armon K, Poland F, Macgregor AJ, Smith TO (2014). Physiotherapy and occupational therapy interventions for people with benign joint hypermobility syndrome: a systematic review of clinical trials. Disabil Rehabil.

[13] Palmer S, Bailey S, Barker L, Barney L, Elliott A (2014). The effectiveness of therapeutic exercise for joint hypermobility syndrome: a systematic review. Physiotherapy..

[14] Warby SA, Pizzari T, Ford JJ, Hahne AJ, Watson L (2014). The effect of exercise-based management for multidirectional instability of the glenohumeral joint: a systematic review. J Shoulder Elb Surg.

[15] Engelbert RH, Juul-Kristensen B, Pacey V, de Wandele I, Smeenk S, Woinarosky N, Sabo S, Scheper MC, Russek L, Simmonds JV (2017). The evidence-based rationale for physical therapy treatment of children, adolescents, and adults diagnosed with joint hypermobility syndrome/hypermobile Ehlers Danlos syndrome. Am J Med Genet C Semin Med Genet.

[16] Warby SA, Ford JJ, Hahne AJ, Watson L, Balster S, Lenssen R, Pizzari T (2018). Comparison of 2 exercise rehabilitation programs for multidirectional instability of the glenohumeral joint: a randomized controlled trial. Am J Sports Med.

[17] Ingwersen KG, Jensen SL, Sorensen L, Jorgensen HR, Christensen R, Sogaard K, Juul-Kristensen B (2017). Three months of progressive high-load versus traditional low-load strength training among patients with rotator cuff tendinopathy: primary results from the double-blind randomized controlled RoCTEx trial. Orthop J Sports Med.

[18] Eshoj HR, Rasmussen S, Frich LH, Hvass I, Christensen R, Boyle E, Jensen SL, Søndergaard J, Søgaard K, Juul-Kristensen B (2020). Neuromuscular exercises improve shoulder function more than standard care exercises in patients with a traumatic anterior shoulder dislocation: a randomized controlled trial. Orthop J Sports Med.

[19] Laudner KG, Metz B, Thomas DQ (2013). Anterior glenohumeral laxity and stiffness after a shoulder-strengthening program in collegiate cheerleaders. J Athl Train.

[20] Couppe C, Kongsgaard M, Aagaard P, Hansen P, Bojsen-Moller J, Kjaer M, Magnusson SP (2008). Habitual loading results in tendon hypertrophy and increased stiffness of the human patellar tendon. J Appl Physiol (1985).

[21] Rombaut L, Malfait F, De Wandele I, Mahieu N, Thijs Y, Segers P, De Paepe A, Calders P (2012). Muscle-tendon tissue properties in the hypermobility type of Ehlers-Danlos syndrome. Arthritis Care Res (Hoboken)..

[22] Liaghat B, Skou ST, Jørgensen U, Sondergaard J, Søgaard K, Juul-Kristensen B (2020). Heavy shoulder strengthening exercise in people with hypermobility spectrum disorder (HSD) and long-lasting shoulder symptoms: a feasibility study. Pilot Feasibility Stud.

[23] Thabane L, Thomas T, Ye C, Paul J (2008). Posing the research question: not so simple. Can J Anesthesia.

[24] Bandholm T, Christensen R, Thorborg K, Treweek S, Henriksen M (2017). Preparing for what the reporting checklists will not tell you: the PREPARE Trial guide for planning clinical research to avoid research waste. Br J Sports Med.

[25] Chan A-W, Tetzlaff JM, Altman DG, Laupacis A, Gøtzsche PC, Krleža-Jerić K, Hróbjartsson A, Mann H, Dickersin K, Berlin JA, Doré CJ, Parulekar WR, Summerskill WSM, Groves T, Schulz KF, Sox HC, Rockhold FW, Rennie D, Moher D (2013). SPIRIT 2013 statement: defining standard protocol items for clinical trials. Ann Intern Med.

[26] Hoffmann TC, Glasziou PP, Boutron I, Milne R, Perera R, Moher D, Altman DG, Barbour V, Macdonald H, Johnston M, Lamb SE, Dixon-Woods M, McCulloch P, Wyatt JC, Chan A-W, Michie S (2014). Better reporting of interventions: template for intervention description and replication (TIDieR) checklist and guide. BMJ.

[27] Slade SC, Dionne CE, Underwood M, Buchbinder R, Beck B, Bennell K, Brosseau L, Costa L, Cramp F, Cup E, Feehan L, Ferreira M, Forbes S, Glasziou P, Habets B, Harris S, Hay-Smith J, Hillier S, Hinman R, Holland A (2016). Consensus on Exercise Reporting Template (CERT): modified Delphi study. Phys Ther.

[28] Toigo M, Boutellier U (2006). New fundamental resistance exercise determinants of molecular and cellular muscle adaptations. Eur J Appl Physiol.

[29] Malfait F, Francomano C, Byers P, Belmont J, Berglund B, Black J, Bloom L, Bowen JM, Brady AF, Burrows NP, Castori M, Cohen H, Colombi M, Demirdas S, De Backer J, De Paepe A, Fournel-Gigleux S, Frank M, Ghali N, Giunta C (2017). The 2017 international classification of the Ehlers-Danlos syndromes. Am J Med Genet C Semin Med Genet..

[30] Hakim AJ, Grahame R (2003). A simple questionnaire to detect hypermobility: an adjunct to the assessment of patients with diffuse musculoskeletal pain. Int J Clin Pract.

[31] The Danish Rheumatism Association. Treatment of hypermobility - prevention of pain and injuries. https://www.gigtforeningen.dk/viden-om-gigt/diagnoser/hypermobilitet/behandling-af-hypermobilitet/. Accessed 9 May 2020.

[32] Arthritis Research UK. Joint hypermobility 2015. Available from: https://www.versusarthritis.org/media/1255/joint-hypermobility-information-booklet.pdf. Accessed 9 May 2020.

[33] Ross S, Grant A, Counsell C, Gillespie W, Russell I, Prescott R (1999). Barriers to participation in randomised controlled trials: a systematic review. J Clin Epidemiol.

[34] Watson L, Balster S, Lenssen R, Hoy G, Pizzari T (2018). The effects of a conservative rehabilitation program for multidirectional instability of the shoulder. J Shoulder Elb Surg.

[35] Brzycki M (1993). Strength testing—predicting a one-rep max from reps-to-fatigue. J Phys Educ Recreation Dance.

[36] Moller MB, Kjaer M, Svensson RB, Andersen JL, Magnusson SP, Nielsen RH (2014). Functional adaptation of tendon and skeletal muscle to resistance training in three patients with genetically verified classic Ehlers Danlos syndrome. Muscles Ligaments Tendons J.

[37] American College of Sports Medicine (2017). ACSM’s guidelines for exercise testing and prescription. 10th Edition ed.

[38] McClure P, Tate AR, Kareha S, Irwin D, Zlupko E (2009). A clinical method for identifying scapular dyskinesis, part 1: reliability. J Athl Train.

[39] Eshoj H, Bak K, Blond L, Juul-Kristensen B (2017). Translation, adaptation and measurement properties of an electronic version of the Danish Western Ontario Shoulder Instability Index (WOSI). BMJ Open.

[40] Kirkley A, Werstine R, Ratjek A, Griffin S (2005). Prospective randomized clinical trial comparing the effectiveness of immediate arthroscopic stabilization versus immobilization and rehabilitation in first traumatic anterior dislocations of the shoulder: long-term evaluation. Arthroscopy..

[41] Salomonsson B, Ahlstrom S, Dalen N, Lillkrona U (2009). The Western Ontario Shoulder Instability Index (WOSI): validity, reliability, and responsiveness retested with a Swedish translation. Acta Orthop.

[42] Breivik H, Borchgrevink PC, Allen SM, Rosseland LA, Romundstad L, Hals EK, Kvarstein G, Stubhaug A (2008). Assessment of pain. Br J Anaesth.

[43] Donnelly C, Carswell A (2002). Individualized outcome measures: a review of the literature. Can J Occup Ther.

[44] Voermans NC, Knoop H (2011). Both pain and fatigue are important possible determinants of disability in patients with the Ehlers-Danlos syndrome hypermobility type. Disabil Rehabil.

[45] Bentsen BG, Natvig B, Winnem M (1999). Questions you didn’t ask? COOP/WONCA charts in clinical work and research. World Organization of Colleges, Academies and Academic Associations of General Practitioners/Family Physicists. Fam Pract.

[46] Kinnersley P, Peters T, Stott N (1994). Measuring functional health status in primary care using the COOP-WONCA charts: acceptability, range of scores, construct validity, reliability and sensitivity to change. Br J Gen Pract.

[47] Mintken PE, Cleland JA, Whitman JM, George SZ (2010). Psychometric properties of the Fear-Avoidance Beliefs Questionnaire and Tampa Scale of Kinesiophobia in patients with shoulder pain. Arch Phys Med Rehabil.

[48] Janssen MF, Pickard AS, Golicki D, Gudex C, Niewada M, Scalone L, Swinburn P, Busschbach J (2013). Measurement properties of the EQ-5D-5L compared to the EQ-5D-3L across eight patient groups: a multi-country study. Qual Life Res.

[49] Hinz A, Kohlmann T, Stobel-Richter Y, Zenger M, Brahler E (2014). The quality of life questionnaire EQ-5D-5L: psychometric properties and normative values for the general German population. Qual Life Res.

[50] Skare O, Liavaag S, Reikeras O, Mowinckel P, Brox JI (2013). Evaluation of Oxford instability shoulder score, Western Ontario shoulder instability index and Euroqol in patients with SLAP (superior labral anterior posterior) lesions or recurrent anterior dislocations of the shoulder. BMC Res Notes.

[51] Kamper SJ, Ostelo RW, Knol DL, Maher CG, de Vet HC, Hancock MJ (2010). Global Perceived Effect scales provided reliable assessments of health transition in people with musculoskeletal disorders, but ratings are strongly influenced by current status. J Clin Epidemiol.

[52] Ingelsrud LH, Terwee CB, Terluin B, Granan LP, Engebretsen L, Mills KAG, Roos EM (2018). Meaningful change scores in the knee injury and osteoarthritis outcome score in patients undergoing anterior cruciate ligament reconstruction. Am J Sports Med.

[53] Ingwersen KG, Christensen R, Sorensen L, Jorgensen HR, Jensen SL, Rasmussen S, Sogaard K, Juul-Kristensen B (2015). Progressive high-load strength training compared with general low-load exercises in patients with rotator cuff tendinopathy: study protocol for a randomised controlled trial. Trials..

[54] Kjaer BH, Magnusson SP, Warming S, Henriksen M, Krogsgaard MR, Juul-Kristensen B (2018). Progressive early passive and active exercise therapy after surgical rotator cuff repair - study protocol for a randomized controlled trial (the CUT-N-MOVE trial). Trials..

[55] Clarsen B, Bahr R, Andersson SH, Munk R, Myklebust G (2014). Reduced glenohumeral rotation, external rotation weakness and scapular dyskinesis are risk factors for shoulder injuries among elite male handball players: a prospective cohort study. Br J Sports Med.

[56] Vafadar AK, Cote JN, Archambault PS (2016). Interrater and intrarater reliability and validity of 3 measurement methods for shoulder-position sense. J Sport Rehabil.

[57] Eshoj H, Rasmussen S, Frich LH, Hvass I, Christensen R, Jensen SL, Sondergaard J, Sogaard K, Juul-Kristensen B (2017). A neuromuscular exercise programme versus standard care for patients with traumatic anterior shoulder instability: study protocol for a randomised controlled trial (the SINEX study). Trials..

[58] Eshoj H, Ingwersen KG, Larsen CM, Kjaer BH, Juul-Kristensen B (2018). Intertester reliability of clinical shoulder instability and laxity tests in subjects with and without self-reported shoulder problems. BMJ Open.

[59] Juul-Kristensen B, Rogind H, Jensen DV, Remvig L (2007). Inter-examiner reproducibility of tests and criteria for generalized joint hypermobility and benign joint hypermobility syndrome. Rheumatology (Oxford).

[60] Nicholson LL, Chan C. The Upper Limb Hypermobility Assessment Tool: A novel validated measure of adult joint mobility. Musculoskelet Sci Pract. 2018;35:38–45. ISSN 2468-7812. 10.1016/j.msksp.2018.02.006. http://www.sciencedirect.com/science/article/pii/S2468781218300468. 10.1016/j.msksp.2018.02.006 29510315

[61] Chester R, Khondoker M, Shepstone L, Lewis JS, Jerosch-Herold C (2019). Self-efficacy and risk of persistent shoulder pain: results of a Classification and Regression Tree (CART) analysis. Br J Sports Med.

[62] Ingelsrud LH, Granan LP, Terwee CB, Engebretsen L, Roos EM (2015). Proportion of patients reporting acceptable symptoms or treatment failure and their associated KOOS values at 6 to 24 months after anterior cruciate ligament reconstruction: a study from the Norwegian Knee Ligament Registry. Am J Sports Med.

[63] van der Linde JA, van Kampen DA, van Beers L, van Deurzen DFP, Saris DBF, Terwee CB (2017). The responsiveness and minimal important change of the Western Ontario Shoulder Instability Index and Oxford Shoulder Instability Score. J Orthop Sports Phys Ther.

[64] Bateman M, Smith BE, Osborne SE, Wilkes SR (2015). Physiotherapy treatment for atraumatic recurrent shoulder instability: early results of a specific exercise protocol using pathology-specific outcome measures. Shoulder Elbow.

[65] Blacknall J, Mackie A, Wallace WA (2014). Patient-reported outcomes following a physiotherapy rehabilitation programme for atraumatic posterior shoulder subluxation. Shoulder Elbow.

[66] Gamble C, Krishan A, Stocken D, Lewis S, Juszczak E, Doré C, Williamson PR, Altman DG, Montgomery A, Lim P, Berlin J, Senn S, Day S, Barbachano Y, Loder E (2017). Guidelines for the content of statistical analysis plans in clinical trials. JAMA..

[67] Moher D, Hopewell S, Schulz KF, Montori V, Gotzsche PC, Devereaux PJ, Elbourne D, Egger M, Altman DG (2010). CONSORT 2010 explanation and elaboration: updated guidelines for reporting parallel group randomised trials. Bmj..

[68] Jarvinen TL, Sihvonen R, Bhandari M, Sprague S, Malmivaara A, Paavola M, Schunemann HJ, Guyatt GH (2014). Blinded interpretation of study results can feasibly and effectively diminish interpretation bias. J Clin Epidemiol.

[69] U.S Food and Drug Administration. What is a serious adverse event?. https://www.fda.gov/safety/reporting-serious-problems-fda/what-serious-adverse-event. Accessed 9 May 2020.

[70] Holmgren T, Hallgren HB, Oberg B, Adolfsson L, Johansson K (2014). Effect of specific exercise strategy on need for surgery in patients with subacromial impingement syndrome: randomised controlled study. Br J Sports Med.

[71] Hulley SB (2007). Designing clinical research: Lippincott Williams & Wilkins.

[72] World Medical Association. World Medical Association Declaration of Helsinki: ethical principles for medical research involving human subjects. JAMA. 2013;310(20):2191-4. PMID: 24141714. 10.1001/jama.2013.281053. https://pubmed.ncbi.nlm.nih.gov/24141714/. 10.1001/jama.2013.281053 24141714

