# Supplementary material for: A randomised controlled trial of heavy shoulder strengthening exercise in patients with hypermobility spectrum disorder or hypermobile Ehlers-Danlos syndrome and long-lasting shoulder complaints: study protocol for the Shoulder-MOBILEX study
Source: Trials. 2020 Dec 1;21:992. doi: 10.1186/s13063-020-04892-0 (PMC7705859; doi:10.1186/s13063-020-04892-0)
Supplement: Supplementary file 2 — Additional file 2. Consensus on Exercise Reporting Template. [file 13063_2020_4892_MOESM2_ESM.pdf]

CERT Question Item #	HEAVY	LIGHT
**Item 1**<br>Detailed description of the type of exercise equipment	The exercises are performed with adjustable dumbbells provided by the project	The exercises are performed with or without a yellow TheraBand resistance band
**Item 2**<br>Detailed description of the qualifications, expertise and or training	The exercises are delivered by the treating physiotherapists, who had undergone approximately 3 hours of education in the exercise programme	
**Item 3**<br>Describe whether exercises are performed individually or in groups	The exercises are performed individually	
**Item 4**<br>Describe whether exercises are supervised or unsupervised, how they are delivered	The exercises are supervised twice a week and performed at home once a week. Duration of first supervised session is 60 minutes, and the duration is 30 minutes for the following sessions	The exercises are performed three times a week at home. Participants will receive a face-to-face introduction before initiating the programme, and supervision at Week 5 and Week 11 when they start with new exercises
**Item 5**<br>Detailed description of how adherence to exercise is measured and reported	Patient adherence is measured using an exercise logbook and reported in % of total exercise sessions (100% equals full adherence corresponding to 48 exercise sessions)	
**Item 6**<br>Detailed description of motivation strategies	Patients received supervised exercises two out of three times per week and were encouraged with a detailed description of the strengthening programme	Patients received three physiotherapy sessions during the exercise programme and were encouraged with a detailed description of the strengthening programme
**Item 7a**<br>Detailed description of the decision rule(s) for determining exercise progression	Exercise progression was applied when the patient could perform more than two extra repetition maximum without pain flare or obvious signs of scapula instability	Exercise progression was predetermined and therefore there were no decision rules for determining exercise progression
**Item 7b**<br>Detailed description of how the exercise program was progressed	The exercise programme was progressed by increasing load and partly by increasing volume/intensity (Supplementary file 3)	The exercise programme was progressed by changing from isometric exercises to dynamic exercises (Supplementary file 3)
**Item 8**<br>Detailed description of each exercise to enable replication	The exercise programme included five exercises identified to target scapular and rotator cuff muscles and tendons:<br><br>Exercise 1) side lying external rotation (ER) in neutral<br>Exercise 2) prone horizontal abduction<br>Exercise 3) prone ER at 90°<br>Exercise 4) supine scapular protraction<br>Exercise 5) standing shoulder elevation in scapular plane	The exercise programme included nine exercises identified to target scapular and rotator cuff muscles and tendons:<br><br>Phase 1<br>Isometric<br>Exercise 1) standing posture correction<br><br>Phase 2<br>Isometric<br>Exercise 2) standing shoulder abduction against a wall<br>Exercise 3) standing shoulder external rotation against a wall with 90° flexion at elbow joint<br>Exercise 4) standing shoulder internal rotation against a wall with 90° flexion at elbow joint<br>Exercise 5) standing weightbearing in the shoulders against a table<br><br>Phase 3<br><br>Dynamic<br>Exercise 6) standing shoulder abduction with resistance band<br>Exercise 7) standing shoulder external rotation with resistance band<br>Exercise 8) standing shoulder internal rotation with resistance band<br>Exercise 9) four-point kneeling with single arm raising

**Item 9**<br>Detailed description of any home-based programme component	There was no difference between the exercise programme performed at home and with the treating physiotherapist. The detailed description of each exercise is specified in item 8 for both groups	
**Item 10**<br>Describe whether there are any non-exercise components	Non-exercise components included general advice about management of load and pain (Supplementary 4 and 5)	
**Item 11**<br>Describe the type and number of adverse events that occur during exercise	Any adverse events that occur during exercise will be registered in an exercise logbook and response to a weekly questionnaire and may involve muscle soreness, pain flare and episodes of subluxation or dislocation	
**Item 12**<br>Describe the setting in which the exercises are performed	The exercises are performed individually in an undisturbed clinical environment and at home	The exercises are performed individually at home
**Item 13**<br>Detailed description of the exercise intervention	The first 3 weeks include a familiarisation period progressing from three sets of 50% with 10 repetition maximum (RM) in the first week to 70% in the second week and 90% in the third week. The following 6 weeks (Weeks 4-9) include progressive heavy strengthening exercises with three sets of 10 RM, and from Weeks 10-15, the exercise load is at four sets of 8 RM. During the entire intervention period, the exercises are performed with a 3 sec concentric and a 3 sec eccentric phase without any isometric hold	The first 4 weeks include a familiarisation period, only consisting of exercise 1 with one set of 10 repetitions. The following 6 weeks (Weeks 5-10) consist of isometric exercises (Exercises 2-5) with 2 sets of 10 repetitions. From Weeks 11-13, the exercises are a mixture of isometric and dynamic (Exercises 2-9) with one set of 10 repetitions of each exercise and from Weeks 14-16, the exercises are dynamic (Exercises 6-9) with 2 sets of 10 repetitions. The isometric hold in the isometric exercises (Exercises 2-5) was 2-3 sec and 10 sec (Exercise 1). There is a 30 sec break in between exercise sets. The resistance band resistance (length of the yellow light band) was managed by the patient alone
**Item 14a**<br>Describe whether the exercises are generic (one size fits all or tailored)	The exercises are tailored to the individual	The exercises are tailored to the individual
**Item 14b**<br>Detailed description of how the exercises are tailored to the individual	The exercises are tailored by adjusting exercise load to match the individual level of repetition maximum and can be modified in case of symptoms or pain flares.<br>The load can be adjusted by decreasing load, reducing range of motion, reducing sets and repetitions, reducing number of exercise sessions per week, and excluding provoking exercises	The exercises are tailored to the individual, since the participants for isometric exercises can adjust the intensity, and for dynamic exercises by adjusting the length of the yellow light resistance band.<br>The load can be modified in case of symptom or pain flares. The load can be reduced by applying less pressure in isometric exercises and adjusting the length of the resistance band. Other options are reducing range of motion, reducing sets and repetitions, reducing number of exercise sessions per week, and excluding provoking exercises
**Item 15**<br>Describe the decision rule for determining the starting level	The starting level is determined by determining one repetition maximum (1RM) in each exercise at the first supervised session. Later, the load will continuously be adjusted to the increase in capabilities of the individual	The starting level is self-determined by the patient; isometric start level is 30-50% of maximal voluntary contraction; dynamic exercises are adjusted so the participant does not experience fatigue
**Item 16a**<br>Describe how adherence or fidelity is assessed/measured	Patient adherence will be measured with an exercise logbook and a weekly online questionnaire	
**Item 16b**<br>Describe the extent to which the intervention was delivered as planned	n/a	n/a
