# Supplementary material for: A randomised controlled trial of heavy shoulder strengthening exercise in patients with hypermobility spectrum disorder or hypermobile Ehlers-Danlos syndrome and long-lasting shoulder complaints: study protocol for the Shoulder-MOBILEX study
Source: Trials. 2020 Dec 1;21:992. doi: 10.1186/s13063-020-04892-0 (PMC7705859; doi:10.1186/s13063-020-04892-0)
Supplement: Supplementary file 3 — Additional file 3 HEAVY exercise manual. [file 13063_2020_4892_MOESM3_ESM.pdf]

EXERCISE MANUAL

YOUR EXERCISE PROGRAMME AND
PAIN MANAGEMENT GUIDELINES

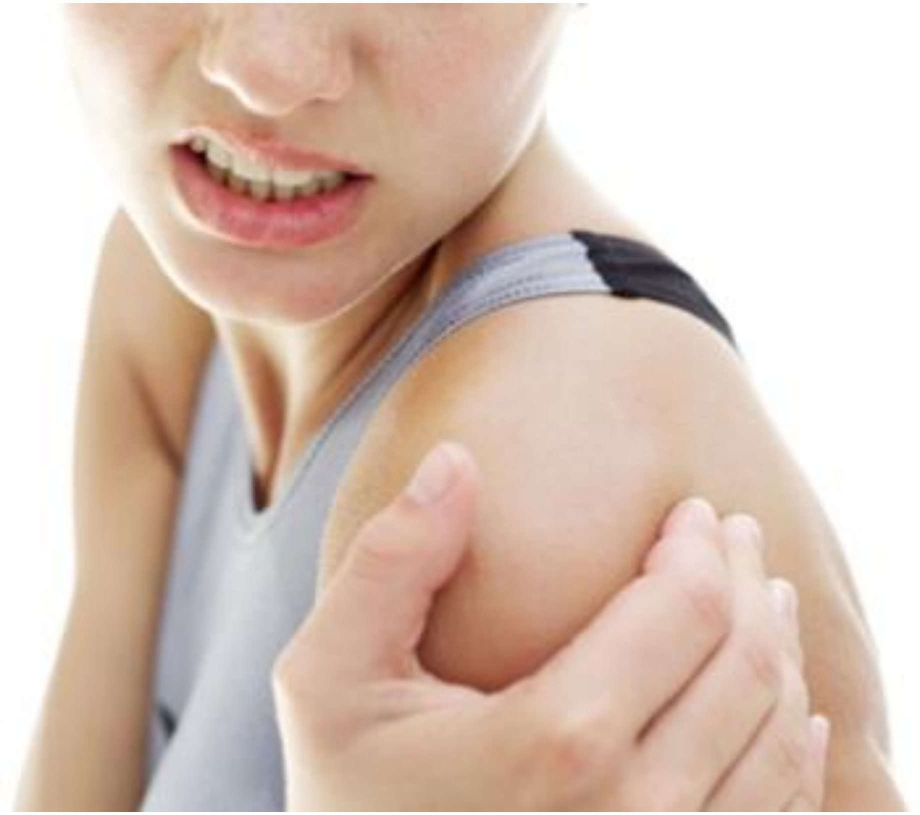

PROJECT MANAGER: BEHNAM LIAGHAT
PHYSIOTHERAPIST, SPECIALIST, MSC.

PHD FELLOW, THE RESEARCH UNIT FOR MUSCULOSKELETAL FUNCTION AND PHYSIOTHERAPY, DEPARTMENT OF SPORTS SCIENCE AND CLINICAL BIOMECHANICS,
UNIVERSITY OF SOUTHERN DENMARK
T. +45 26 82 68 01, bliaghat@health.sdu.dk

YOUR PHYSIOTHERAPIST IS: ________________________

GENERAL INFORMATION ABOUT THE EXERCISE PROGRAMME

PURPOSE

The programme is designed to increase strength in the shoulder and in the muscles around the shoulder blade, so you can better control movements in everyday life. The first 4 weeks are a preparatory phase, in which the load gradually increases. The strengthening programme will then begin.

GENERAL RECOMMENDATIONS

Exercising regularly is an important component of a healthy lifestyle, and there is no reason why people with hypermobile joints should not exercise. But if you find that certain sports or exercise activities provoke your pain, you should stop these activities until you know why your symptoms occur. With the right strengthening exercises, it may be possible to return to the specific activities without increasing symptoms. Controlled strengthening exercises and correction exercises should therefore be good for the body.

- Make sure you keep your muscles strong. This applies to the muscles around and between the shoulder blades as well as the muscles in the front of the neck.
- Be aware of your posture to avoid straining joints in extreme positions. This applies both at work, when engaging in hobbies and at rest.
- Avoid twisting the back or working with a bent back.
- Think about your work routines – make sure you have variation.
- Be conscious of lifting in a way that does not provoke your symptoms unnecessarily.
- If a movement or activity gives you symptoms, it is a good idea to measure how long it takes for you to perform the activity without symptoms. Shorten the time by 25% and gradually increase again as symptoms take longer to appear.

CONTROLLED MOVEMENTS

All exercises must be carried out in a controlled way and with controlled movements. Pay attention to the quality of the exercises, including checking your shoulder blades in all exercises so that the desired effect can be achieved. Focus points for achieving this are set out in the exercise description and your physiotherapist will help you get started. You should perform the session at home at a time during the day when you can perform the programme without being disturbed.

EXERCISE LOAD

You must adjust the load to suit the given repetitions, so that you cannot take more repetitions with the given load.

The load for the 16 weeks of training looks like

Week 1:	50% of 10 RM	3 x 10
Week 2:	70% of 10 RM	3 x 10
Week 3:	90% of 10 RM	3 x 10
Weeks 4-9:	10 RM	3 x 10
Weeks 10-15:	8 RM	4 x 8
Week 16:	70% of 8 RM	4 x 8

WARMING UP

Always start the exercise programme by warming up your shoulders by performing the exercises without load. Make 10 repetitions of each exercise before then proceeding with adding the load.

PAIN DURING EXERCISE

You can experience slight pain in the shoulder during exercise. It is normal and acceptable. It is important that you are aware that pain should not rise above 5 on the 0-10 Numerical Pain Rating Scale (0 no pain, 10 extreme pain). A level of 5 is equivalent to that which you can tolerate without feeling that the pain is directly harmful.

Any experience of pain should drop to the level it was before the exercise session began within 30 minutes/before the next exercise session. In addition, the pain should not increase over the coming days. If the pain does not subside or gets worse, the load should be reduced over the next 3-4 exercise sessions. If the pain persists, please contact your physiotherapist (front page information). If you are taking painkillers, the pain level may generally be lower, but the same guidelines apply.

HOW DOES THE PROGRAMME WORK?

Exercising the muscles around your shoulder blade is important as it is directly related to your shoulder joint and is essential for your shoulder function. You must perform the following five strengthening exercises three times a week. The exercises must be carried out with good quality and control and at a smooth and slow pace to get the optimal benefit from the exercises. Twice a week you will have to do the exercises together with a physiotherapist, and once a week you will have to do the exercises at home.

You must perform 3-4 sets of 8-10 repetitions of each exercise. When you can perform more repetitions than the specified number in each set of a given exercise, the load should be increased in consultation with the physiotherapist. The first 4 weeks will be a slow preparatory phase for you to get used to the strengthening exercises, so the load will increase every week until you can do just 10 repetitions in each set.

After each workout, fill out your training logbook.

EXERCISE 1: SIDE-LYING OUTWARD ROTATION

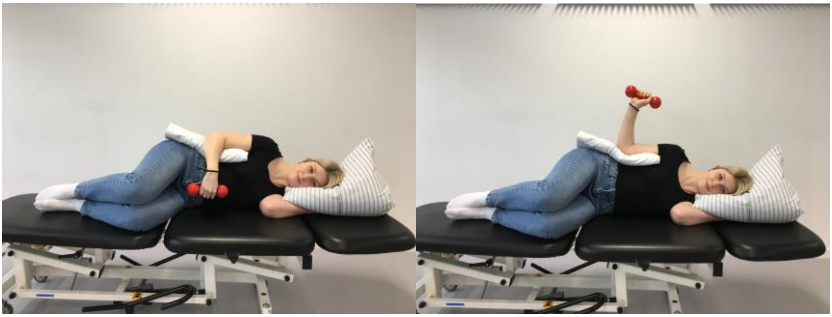

EXECUTION

Lie on the side with flexed hips and knees.

- Place the lower arm on the supporting surface, and rest the head on a pillow
- Bend the upper arm (the exercising arm) with the elbow at 90 degrees
- Place a pillow between the upper arm and upper body
- Hold the dumbbell in the hand
- Rest the hand on the abdomen
- Stabilize the shoulder blade by lifting the shoulder slightly towards the ear, while moving the shoulder slightly down/back towards the spine
- Upwardly rotate the arm while holding the upper arm close to the body
- Slowly lower the arm

Weeks 1-9: Complete 3 sets of 10 repetitions with 60 seconds break between each set.
Week s10-16: Complete 4 sets of 8 repetitions with 90 seconds break between each set.

Focus: *Avoid the shoulder blade moving up to the ear or back and forth*

EXERCISE 2: PRONE ARM RAISE WITH OUTWARD ROTATION

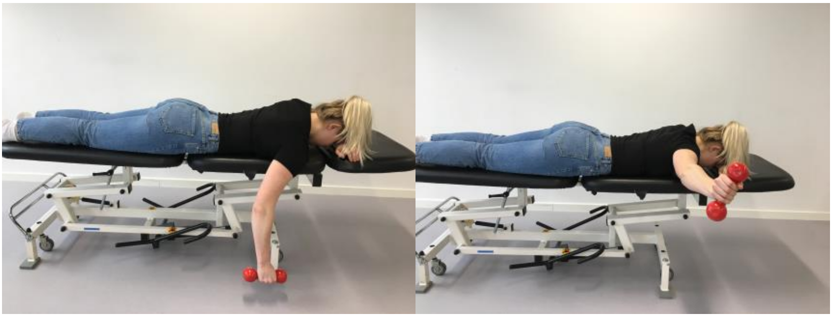

EXECUTION

- Lie prone on a table with the arm hanging towards the floor and the shoulder resting at 90 degrees of flexion
- Hold the dumbbell in the hand
- Stabilize the shoulder blade by lifting the shoulder slightly towards the ear, while moving the shoulder slightly down/back towards the spine
- Lift the arm straight out to the side up to horizontal as you turn the arm/hand outwards
- When you reach the horizontal position, outwardly rotate the hand further
- Lower the arm slowly while returning it back to the starting position

Weeks 1-9: Complete 3 sets of 10 repetitions with 60 seconds break between each set.
Weeks 10-16: Complete 4 sets of 8 repetitions with 90 seconds break between each set.

Focus: *Retract and squeeze together the shoulder blades when you perform the movement*

EXERCISE 3: SEATED ARM LIFT OBLIQUELY FORWARD

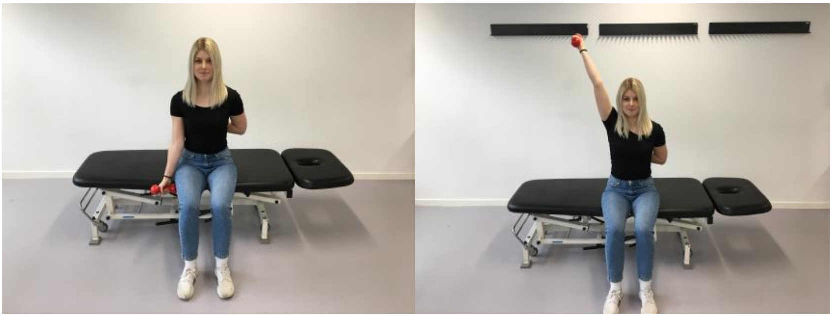

EXECUTION

-Sit on a chair without a backrest, with a straight back and a hip width between your feet
-Hold the dumbbell in the hand and turn the shoulder so the thumb points obliquely to the side.
- Stabilize the shoulder blade by placing the index finger and middle finger of the opposite hand behind the back underneath the shoulder blade and then moving the bony point backwards and away from the fingers
-Activate the deep stabilizing back muscles by exhaling with the mouth closed (and the teeth clenched together).
-Lift the arm obliquely forward and up
-Lower the arm slowly

Weeks 1-9: Complete 3 sets of 10 repetitions with 60 seconds break between each set.
Week s10-16: Complete 4 sets of 8 repetitions with 90 seconds break between each set.

Focus: *Control the movement by placing the opposite hand behind the back underneath the shoulder blade. Notice that the shoulder blade is constantly stable against the rib cage*

EXERCISE 4: PRONE OUTWARD ROTATION WITH SHOULDER AND ELBOW AT 90°

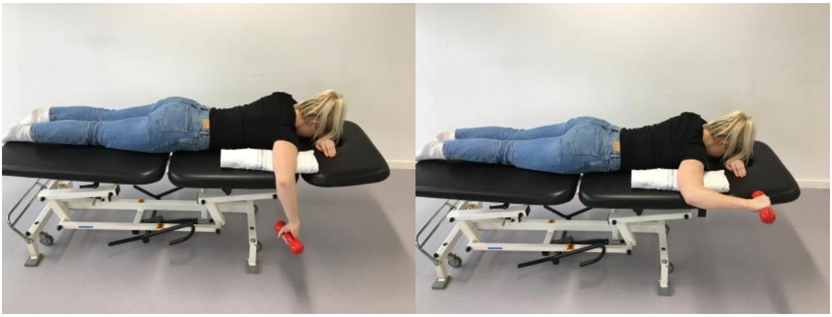

EXECUTION

- Lie prone on a table
- Place the exercising arm at 90 degrees of abduction, the upper arm supported by the table
- Bend the arm 90 degrees at the elbow joint and let the forearm hang vertically down.
- Hold the dumbbell in the hand
- Rest the head on the opposite arm or turn the head to the side
- Stabilize the shoulder blade by lifting the shoulder slightly towards the ear, while moving the shoulder slightly down/back towards the spine
- Turn the forearm up towards the ceiling
- Lower it again slowly

Weeks 1-9: Complete 3 sets of 10 repetitions with 60 seconds break between each set.
Weeks 10-16: Complete 4 sets of 8 repetitions with 90 seconds break between each set.

Focus: *Control the movement by placing the opposite hand behind the back underneath the shoulder blade. Emphasise that the shoulder blade is approaching the fingertips*

EXERCISE 5: SUPINE PUSH WITH ARM

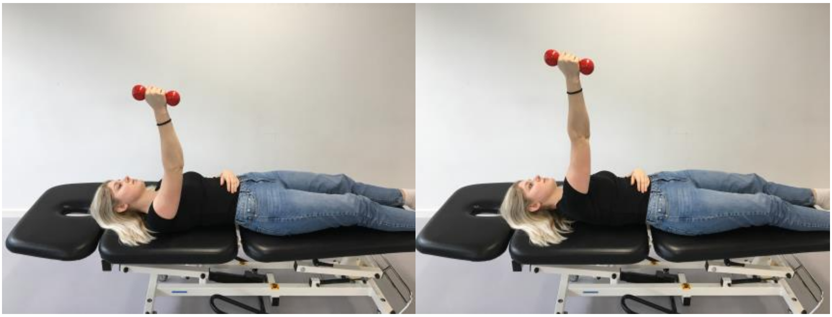

EXECUTION

- Lie on the floor or on a table
- Flex the shoulder to 90 degrees with the hand pointing up towards the ceiling
- Hold the dumbbell in the hand
- Rest the head on the supporting surface
- Keep your head and back on the supporting surface
- Press the stretched arm up towards the ceiling so that the arm is extended, and the shoulder is no longer supported
- Slowly lower the shoulder again so it comes down to touch the supporting surface
- Make sure that the shoulder is not pulled up against the ear and that the arm is pressed straight up towards the ceiling

Weeks 1-9: Complete 3 sets of 10 repetitions with 60 seconds break between each set.
Weeks 10-16: Complete 4 sets of 8 repetitions with 90 seconds break between each set.

Focus: *Put the hand of the opposite arm relaxed on the shoulder and feel that the shoulder is not moving up towards the ear.*

This leaflet was made by Behnam Liaghat for research purposes.
