# Supplementary material for: A randomised controlled trial of heavy shoulder strengthening exercise in patients with hypermobility spectrum disorder or hypermobile Ehlers-Danlos syndrome and long-lasting shoulder complaints: study protocol for the Shoulder-MOBILEX study
Source: Trials. 2020 Dec 1;21:992. doi: 10.1186/s13063-020-04892-0 (PMC7705859; doi:10.1186/s13063-020-04892-0)
Supplement: Supplementary file 4 — Additional file 4. LIGHT exercise manual. [file 13063_2020_4892_MOESM4_ESM.pdf]

EXERCISE MANUAL

YOUR EXERCISE PROGRAMME AND
PAIN MANAGEMENT GUIDELINES

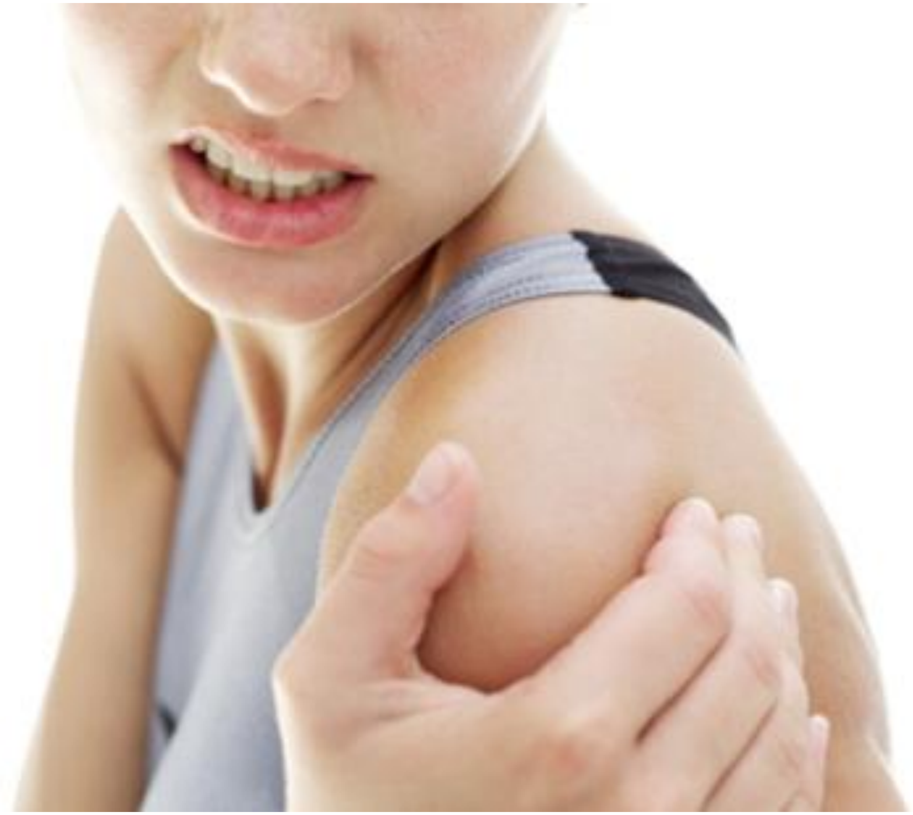

PROJECT MANAGER: BEHNAM LIAGHAT
PHYSIOTHERAPIST, SPECIALIST, MSC.

PHD FELLOW, THE RESEARCH UNIT FOR MUSCULOSKELETAL FUNCTION AND PHYSIOTHERAPY, DEPARTMENT OF SPORTS SCIENCE AND CLINICAL BIOMECHANICS,
UNIVERSITY OF SOUTHERN DENMARK
T. +45 26 82 68 01, bliaghat@health.sdu.dk

YOUR PHYSIOTHERAPIST IS: ________________________

GENERAL INFORMATIONON ABOUT THE PROGRAMME

PURPOSE

The programme is designed to increase strength in the shoulder and in the muscles around the shoulder blade, so you can better control movements in everyday life.

GENERAL RECOMMENDATIONS

Exercising regularly is an important component of a healthy lifestyle, and there is no reason why people with hypermobile joints should not exercise. But if you find that certain sports or exercise activities provoke your pain, you should stop these activities until you know why your symptoms occur. With the right strength exercises, it may be possible to return to the specific activities without increasing symptoms. Controlled strengthening exercises and correction exercises should therefore be good for the body.

- Make sure you keep your muscles strong. This applies to the muscles around and between the shoulder blades as well as the muscles in the front of the neck.
- Be aware of your posture to avoid straining joints in extreme positions. This applies both at work, when engaging in hobbies and at rest.
- Avoid twisting the back or working with a bent back.
- Think about your work routines – make sure you have variation.
- Be conscious of lifting in a way that does not provoke your symptoms unnecessarily.
- If a movement or activity gives you symptoms, it is a good idea to measure how long it takes for you to perform the activity without symptoms. Shorten the time by 25% and gradually increase again as symptoms take longer to appear.

CONTROLLED MOVEMENTS

All exercises must be carried out in a controlled way and with controlled movements. Pay attention to the quality of the exercises, including checking the position of your shoulder blade in all exercises, so that the desired effect can be achieved. Focus points for achieving this are set out in the exercise description and your physiotherapist will help you get started. You should perform the session at home at a time during the day when you can perform the programme without being disturbed.

EXERCISE LOAD

You need to adjust the exercise level so that it allows you to complete 10 repetitions, but without causing fatigue.

The load for the 16 weeks of exercises is structured as follows:

Week 1-4	Position correction	1 x 10
Weeks 5-10	Static	2 x 10
Weeks 11-13	Static +dynamic	1 x 10 of each exercise
Weeks 14-16	Dynamic	2 x 10

PAIN DURING EXERCISE

You can experience slight pain in the shoulder during exercise. It is normal and acceptable. It is important that you are aware that pain should not rise above 5 on the 0-10 Numerical Pain Rating Scale (0 no pain, 10 extreme pain). A level of 5 is equivalent to tolerating the pain without feeling that the pain is directly harmful.

An experience of pain should drop to the same level that it was before the exercise session began within 30 minutes/before the next exercise session. In addition, the pain should not increase over the coming days. If the pain does not subside or gets worse, the load should be reduced over the next 3-4 exercise sessions. If the pain persists, please contact your physiotherapist (front page information). If you are taking painkillers, the pain level may generally be lower, but the same guidelines apply.

HOW DOES THE PROGRAMME WORK?

Exercising the muscles around your shoulder blade is important as it is directly related to your shoulder joint and is essential for your shoulder function. The following static and dynamic exercises should be performed three times weekly. The exercises must be carried out with good quality and control and at a smooth and slow pace to get the optimal benefit from the exercises.

In all the exercises, it is important that you straighten the back, push the chest forward and retract the shoulder blades a little at the back.

After each workout, fill out your exercise logbook.

EXERCISE 1: POSTURE UPRIGHT

PERFORMED WEEK 1-4

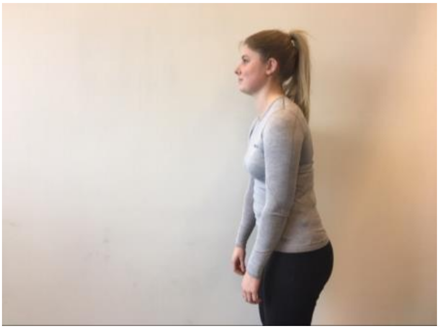

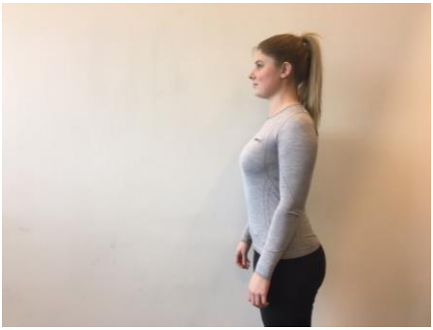

EXECUTION

Stand in a relaxed position.

- Try to increase the distance between the sternum and the navel until the back is upright.
- With a straight back, try to slowly retract the shoulder blades
- Hold this position while taking 3 deep breaths (about 10 sec.)
- Then relax completely and leave the shoulders/back in a relaxed position.

If you experience pain, adjust the degree of movement, so you only experience minimal pain (i.e. a maximum of 5 on the Numerical Pain Rating Scale) before it then disappears.

Complete 1 set of 10 repetitions.

EXERCISE 2: STATIC EXERCISE, ABDUCTION

PERFORMED 2 X 10 IN WEEKS 5-10 AND 1 X 10 IN WEEKS 11-13

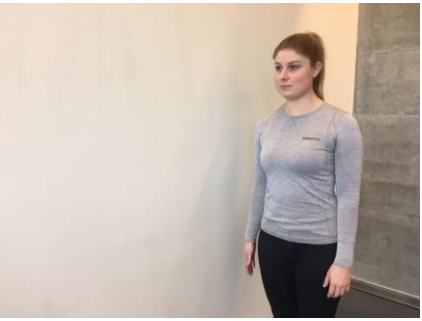
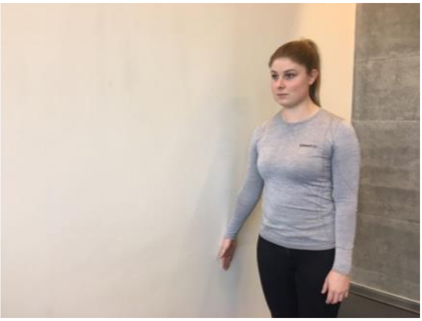

EXECUTION

Stand with the feet a hip-width apart and the shoulder against the door/wall.

Start by standing with a straight back and retract the shoulder blades

- Press the back of the hand outwards towards the door/wall
- Press with approximately 30-50% intensity
- Hold the pressure for 2-3 seconds and feel the activity in the shoulder muscles.

Complete 2 sets of 10 repetitions.
Take about 30 seconds break between each set.

EXERCISE 3: STATIC EXERCISE OUTWARD ROTATION

PERFORMED 2 X 10 IN WEEKS 5-10 AND 1 X 10 IN WEEKS 11-13

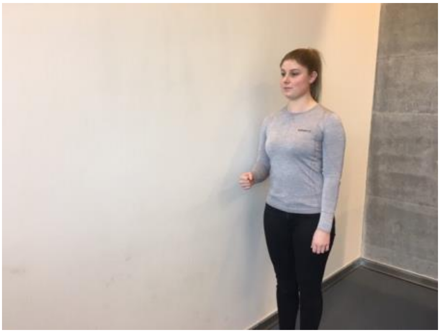
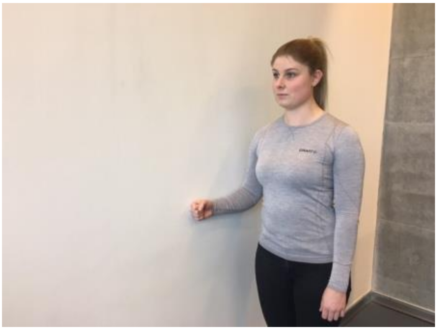

EXECUTION

Stand with the shoulder against the door/wall.

- Start by standing with an upright back and retract the shoulder blades.
- Bend the elbow at 90 degrees, with the thumb pointing towards the ceiling
- Now press the back of the hand against the wall
- Press with approximately 30-50% intensity
- Hold the pressure for 2-3 seconds and feel the activity in the shoulder muscles

Complete 2 sets of 10 repetitions
Take about 30 seconds break between each set.

EXERCISE 4: STATIC EXERCISE INWARD ROTATION

PERFORMED 2 X 10 IN WEEKS 5-10 AND 1 X 10 IN WEEKS 11-13

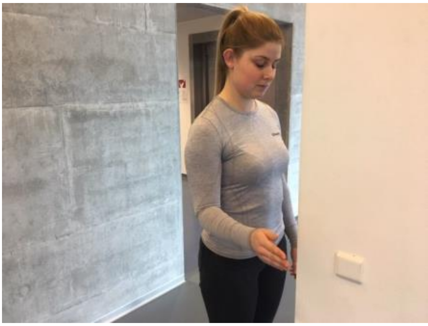

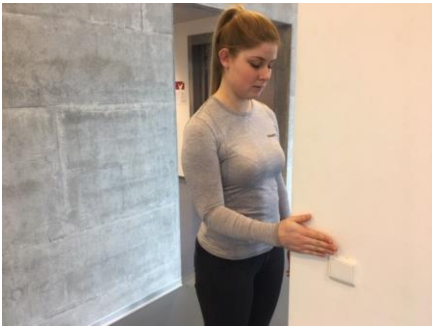

EXECUTION

Stand facing a cupboard or a doorframe.

- Start by standing with a straight back and retract the shoulder blades
- Bend the elbow at 90 degrees, with the thumb pointing towards the ceiling
- Now press the palm of the hand against the cupboard/doorframe
- Press with approximately 30-50% intensity
- Hold the pressure for 2-3 seconds and feel the activity in the shoulder muscles

Complete 2 sets of 10 repetitions
Take about 30 seconds break between each set.

EXERCISE 5: STATIC EXERCISE PUSH UP

PERFORMED 2 X 10 IN WEEKS 5-10 AND 1 X 10 IN WEEKS 11-13

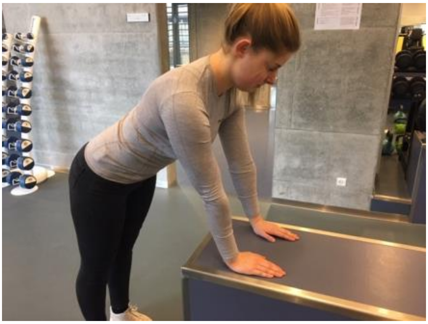

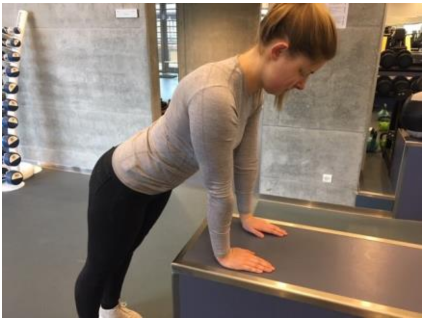

EXECUTION

Stand next to a table.

- Contract the muscles around the shoulder blades
- Lean forward a little and put both hands on the table with arms straight and elbows locked
- Now lean forward so that the weight is transferred to both arms
- Hold the pressure 2-3 seconds

You may put as much pressure on the arms as you feel your pain allows.

Complete 2 sets of 10 repetitions.
Take about 30 seconds break between each set.

EXERCISE 6: DYNAMIC - ABDUCTION

PERFORMED 1 X 10 IN WEEKS 11-13 AND 2 X 10 IN WEEKS 14-16

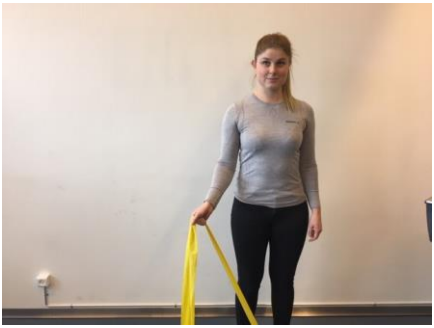

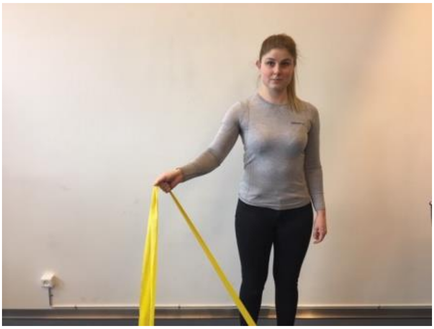

EXECUTION

Stand holding an elastic band

- Place the elastic band under the foot and hold the elastic band with the opposite hand (exercise arm)
- Stand with an upright back and retract the shoulder blades
- Push the exercise arm obliquely outwards and up towards the ceiling with a straight arm, with the thumb pointing upwards. Stop when the arm is at 45 degrees from the body (as shown in the picture)
- Resist slowly when bringing the arm down again

You should lift the arm sideway towards the shoulder, as much as the symptoms allow - up to a maximum of 45 degrees.
The resistance must be at a level that does not cause fatigue after 10 repetitions.

You can adjust the resistance by changing the length of the elastic band (move the hand along the elastic band, shortening the length to increase resistance, or increasing the length to reduce resistance)

Complete 2 sets of 10 repetitions.
Take about 30 seconds break between each set.

EXERCISE 7: DYNAMIC - OUTWARD ROTATION

PERFORMED 1 X 10 IN WEEKS 11-13 AND 2 X 10 IN WEEKS 14-16

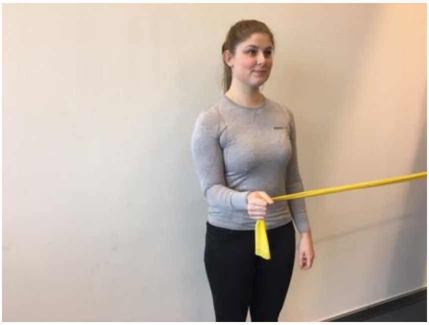
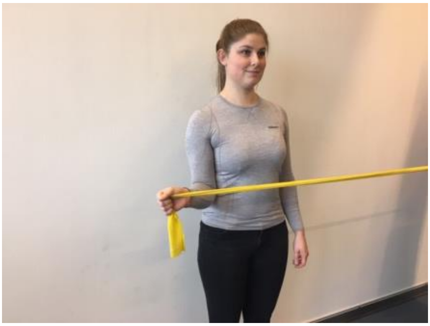

EXECUTION

Stand side on to a door

- Tie the elastic band to the door handle
- Stand side on to the door with the furthest palm facing the door
- Bend the elbow at 90 degrees with the thumb towards the ceiling
- Stand with a straight back and retract the shoulder blades
- Move the forearm outwards at a slow pace, keeping the elbow beside the body/waist
- Resist slowly on the way back

You must move the forearm from neutral position and outwards, as far as you can within the pain limit, but up to a maximum of about 45 degrees.
The resistance must be at a level that does not cause fatigue after 10 repetitions.

You can adjust the resistance by changing the length of the elastic band (move the hand along the elastic band, shortening the length to increase resistance, or increasing the length to reduce resistance)

Complete 2 sets of 10 repetitions.
Take about 30 seconds break between each set.

EXERCISE 8: DYNAMIC - INWARD ROTATION

PERFORMED 1 X 10 IN WEEKS 11-13 AND 2 X 10 IN WEEKS 14-16

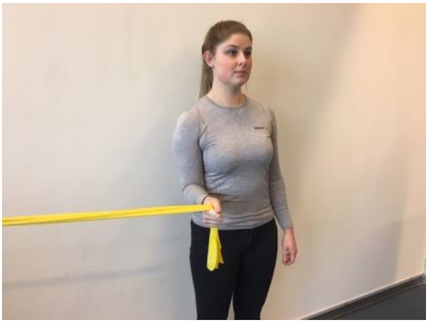
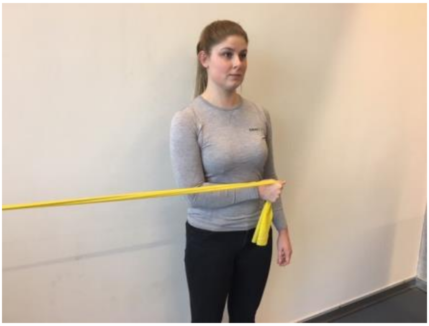

EXECUTION

Stand side on to a door

- Tie the elastic band to the door handle
- Stand side on to the door with the back of the hand facing the door
- Bend the elbow at 90 degrees with the thumb towards the ceiling
- Stand with the back straight and retract the shoulder blades
- Move the forearm inwards at a slow pace keeping the elbow beside the body/waist
- Resist slowly on the way back

You must move the forearm from neutral position and inwards as far as you can within the pain limit.
The resistance must be at a level that does not cause fatigue after 10 repetitions.

You can adjust the resistance by changing the length of the elastic band (move the hand along the elastic band, shortening the length to increase resistance, or increasing the length to reduce resistance).

Complete 2 sets of 10 repetitions.
Take about 30 seconds break between each set.

EXERCISE 9: DYNAMIC – FOUR-POINT KNEELING ARM FLEXION

PERFORMED 1 X 10 IN WEEKS 11-13 AND 2 X 10 IN WEEKS 14-16

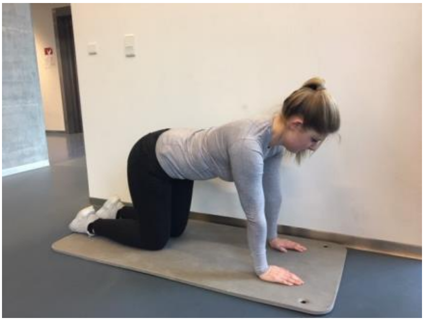

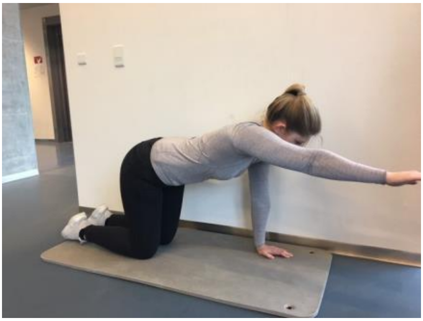

EXECUTION

The exercise must be performed in a four-point kneeling position.

- Ensure the back and shoulder are stable throughout the exercise
- Lift one arm forwards from the floor, keeping the arm straight

Complete 2 sets of 10 repetitions.
Take about 30 seconds of break between each set.

Liaghat et al.
Supplementary file 4

This leaflet was made by Behnam Liaghat for research purposes.
